# Bighorn sheep associations: understanding tradeoffs of sociality and implications for disease transmission

**DOI:** 10.7717/peerj.15625

**Published:** 2023-08-08

**Authors:** Marie I. Tosa, Mark J. Biel, Tabitha A. Graves

**Affiliations:** 1 Northern Rocky Mountain Science Center, U.S. Geological Survey, West Glacier, MT, United States of America; 2 Glacier National Park, National Park Service, West Glacier, MT, United States of America

**Keywords:** Landscape of fear, Many eyes hypothesis, Dilution effect, Respiratory disease, Glacier National Park, *Ovis canadensis*, Contact, Habitat selection, Landscape of peril

## Abstract

Sociality directly influences mating success, survival rates, and disease, but ultimately likely evolved for its fitness benefits in a challenging environment. The tradeoffs between the costs and benefits of sociality can operate at multiple scales, resulting in different interpretations of animal behavior. We investigated the influence of intrinsic (*e.g.*, relatedness, age) and extrinsic factors (*e.g.*, land cover type, season) on direct contact (simultaneous GPS locations ≤ 25 m) rates of bighorn sheep (*Ovis canadensis*) at multiple scales near the Waterton-Glacier International Peace Park. During 2002–2012, male and female bighorn were equipped with GPS collars. Indirect contact (GPS locations ≤ 25 m regardless of time) networks identified two major breaks whereas direct contact networks identified an additional barrier in the population, all of which corresponded with prior disease exposure metrics. More direct contacts occurred between same-sex dyads than female-male dyads and between bighorn groups with overlapping summer home ranges. Direct contacts occurred most often during the winter-spring season when bighorn traveled at low speeds and when an adequate number of bighorn were collared in the area. Direct contact probabilities for all dyad types were inversely related to habitat quality, and differences in contact probability were driven by variables related to survival such as terrain ruggedness, distance to escape terrain, and canopy cover. We provide evidence that probabilities of association are higher when there is greater predation risk and that contact analysis provides valuable information for understanding fitness tradeoffs of sociality and disease transmission potential.

## Introduction

Understanding the underlying mechanisms of social associations can help address questions in evolutionary, behavioral, and infectious disease ecology (Pinter-Wollman et al., 2014; Janousek et al., 2021). Sociality, defined as consistent associations between individuals, can benefit individuals, populations, or species in a challenging environment (Hinde, 1976; Krebs & Davies, 1997; Krause & Ruxton, 2002). Benefits include greater sharing of information, better body condition, increased survival, and higher mating success (Krebs & Davies, 1997; Krause & Ruxton, 2002). Mechanisms for these fitness benefits include improved access to and defense of a resource (space, food, or offspring), increased collective vigilance for predators (the many eyes hypothesis), and dilution of risk of predation with increasing group size (the dilution effect) (Delm, 1990; Rieucau & Martin, 2008) and/or dilution of risk of disease by gaining collective immunity (Plowright et al., 2013). Sociality, however, can also be costly through competition for limited resources, risk of injuries through interactions with aggressive individuals, and transmission and persistence of parasites and infectious diseases (Anderson et al., 1986; Altizer et al., 2003). A balance between these costs, benefits, and selective pressures in a “landscape of peril” ultimately shapes sociality (Doherty & Ruehle, 2020).

Trade-offs between costs and benefits can operate concurrently and at multiple scales (Hinde, 1976; Sih, Hanser & McHugh, 2009; Van der Wal et al., 2016). At the population level, associations can reflect the connectivity among individuals across the landscape and can influence metapopulation dynamics. At a finer scale, the strengths of associations between individuals can reflect dyad and individual characteristics such as personality, genetic relatedness (which can increase indirect fitness), behavior phenomenon such as homophily (*i.e.,* a tendency to interact with individuals of similar age, sex, body size, or social status; McPherson, Smith-Lovin & Cook, 2001), and other intrinsic factors (Hamilton, 1964; Wolf & Weissing, 2012). Finally, at the finest scale, locations of associations can reflect time- and habitat-specific characteristics and other extrinsic factors that relate to access to resources and survival of the individual (Travis, Slobodchikoff & Keim, 1995; Sterck, Watts & Van Schaik, 1997; Naud et al., 2016).

Although the importance of the finest scale, the spatial context of associations, is often acknowledged for understanding factors that shape interactions and the types of behavior they represent (Trillmich & Wolf, 2008; Jacoby & Freeman, 2016), it remains missing from analyses of many social wildlife species (Pinter-Wollman, Fiore & Theraulaz, 2017; Albery et al., 2021). The few studies that have incorporated spatial data have demonstrated the influence of nest structure on the collective foraging performance of ants (Pinter-Wollman, 2015) and the importance of interactions that occur at the periphery of home ranges, which allow individuals to defend their territory (Giuggioli, Potts & Harris, 2011; Spiegel et al., 2018). Fortunately, these data are readily available through proximity-based metrics derived from biologging techniques such as global positioning system (GPS) devices that allow for simultaneous tracking of multiple individuals. Through these proximity-based metrics, GPS devices allow researchers to disentangle an individual’s social behavior with conspecifics from its spatio-temporal distribution in relation to habitat (Webber & Van der Wal, 2018).

Bighorn sheep (*Ovis canadensis*), hereafter bighorn, are a model system to examine social behavior because extensive research exists on habitat selection and disease ecology of bighorn. Although considered secure at global and state scales, local extirpation of herds, mostly due to disease, has occurred throughout their range (Flesch et al., 2022). Bighorn, as social mountain ungulates and habitat specialists, face significant evolutionary pressures from three main factors: competition, predation, and disease. Strong dominance hierarchies within bighorn populations are established and reinforced with intra-sexual competition and may determine reproductive success (Geist, 1971). Males compete with other males through intense dominance interactions before the breeding season and aggressive interactions during the breeding season (Hogg, 1987; Shackleton, Shank & Wikeem, 1999). Predation, especially by mountain lions (*Puma concolor*), accounts for a large portion of bighorn mortality for both male and female bighorn of all age classes (Ross, Jalkotzy & Festa-Bianchet, 1997; Hayes et al., 2000; Rominger et al., 2004; McKinney, Smith & De Vos Jr, 2006). As such, bighorn habitat selection is heavily influenced by variables related to survival, such as distance to escape terrain and canopy cover (DeCesare & Pletscher, 2006). As with most species, multiple diseases can influence bighorn health including gastrointestinal parasites (*e.g.*, *Ostertagia* spp., *Nematodirus* spp., *Cooperia* spp., Becklund & Senger, 1967) and pathogens including *Mycoplasma ovipneumoniae*, *Pasteurellaceae* (*e.g.*, *Mannheimia haemolytica*, *Bibersteinia trehalosi*, *Pasteurella multocida*), and *Anaplasma ovis* (Tibbitts et al., 1992; Besser et al., 2013; Butler et al., 2018). Many of these pathogens have been investigated as the causal agent for respiratory disease, which is transmitted by direct contact and often cited as a significant factor limiting the distribution and abundance of bighorn (Cassirer et al., 2017). Respiratory disease has been implicated in die-offs of up to 90% of exposed bighorn populations (Spraker et al., 1984; Coggins, 1988; Festa-Bianchet, 1988; Besser et al., 2012). Because respiratory disease can have such high mortality rates, disease exposure and disease status of bighorn have been monitored extensively and can provide coarse fitness consequences to sociality. Although considerable research exists on social interactions, habitat selection, and disease ecology of bighorn separately, few studies simultaneously examine the interactions between these selective pressures and few studies address the mechanisms through which bighorn use risky areas where predation may be more likely or the mechanisms by which disease spreads (but see Manlove et al., 2014; Cassirer et al., 2017; Manlove et al., 2017). By studying the associations and the absence of associations between bighorn in the context of multiple selective pressures, we can better understand disease dynamics and conserve wild bighorn populations.

We evaluated associations between male and female bighorn at 3 scales, asking (1) where are the divisions among subpopulations, (2) which intrinsic factors influence frequency of associations between dyads, and (3) when do direct contacts occur and which extrinsic factors influence direct contact structure? We predicted that population structure based on indirect contacts would parallel the structure derived from surveys for diseases transmitted through direct contact (De la Fuente et al., 2007; Ott et al., 2009). For intrinsic factors that influence frequency of direct associations between dyads, we predicted that direct associations would be strongest between genetically related female–female dyads that had high space-use overlap and weakest between unrelated male–female dyads that had low space-use overlap. For timing of contacts, we predicted that more direct contacts would occur when bighorn are most active (*i.e.,* during the day), during the winter for same sex dyads, and during the mating season for male–female dyads. For direct contact locations, we formed multiple hypotheses based on two categories (Table 1): the need for resources and the need for grouping to avoid predation (*i.e.,* survival). For example, we hypothesized that contact locations would be influenced more by survival variables than by resource variables for all dyad types. We predicted that direct contacts would occur more frequently in areas with fewer resources and higher predation risk.

**Table 1:** Extrinsic variables used in habitat use and contacts locations given habitat use models for bighorn sheep in and around Glacier National Park, Montana, USA.

**Variable**	**Units**	**Description**	**Source**	**Res (m)**	**Terms**
**Resources:**					
LC	NA	Land cover: water, barren, ice/snow, developed, scrub/shrub, wetland, grass, agriculture, coniferous forest, deciduous forest, mixed forest	Crown Managers Partnership	30	NA
Elevation	m	Digital elevation model	Crown Managers Partnership	30	L, Q, P
SRI	NA	Solar radiation index as calculated according to Keating et al. 2007	Digital elevation model	30	L, Q
SWE	kg/m^2^	Daily measure of snow water equivalent; amount of water contained within snowpack	Daymet; Oak Ridge National Laboratory	1000	L, P
NDVI	NA	16-day satellite imagery of normalized difference vegetation index	MODIS/ MOD13A1 (Reverb.echo.nasa.gov) Thornton et al. 2014	500	L, P
IRG	NA	Instantaneous rate of green up	NDVI	500	L
d.mlick	m	Distance from known mineral licks	Glacier National Park	30	L, P
d.H_2_O.per	m	Distance to perennial streams and lakes	Crown Managers Partnership	30	L, P
**Survival:**					
Slope	degree	Slope	Digital elevation model	30	L
VRM		Vector ruggedness measure as calculated by Sappington et al. 2007	Digital elevation model	30	L, Q, P
d.esc	m	Distance to escape terrain (slope >27 deg)	Digital elevation model	30	L, P
Canopy	%	Canopy cover	Crown Managers Partnership	30	L, P
**Disturbance:**					
d.road	m	Distance to paved roads	Crown Managers Partnership	30	L, P
d.trail	m	Distance to established hiking trails	National Park Service	30	L, P
d.heli	m	Distance to known helicopter tour routes	National Park Service Personal communication, R. Menicke	30	L, P

**Notes.**

Extrinsic variables used in habitat use and contact locations (given habitat use) models for bighorn sheep in and around Glacier National Park, Montana, USA.

Terms represent transformations of variables used: linear (L), quadratic (Q), and pseudo-threshold (P).

## Materials & Methods

### Study area

**Figure 1:**
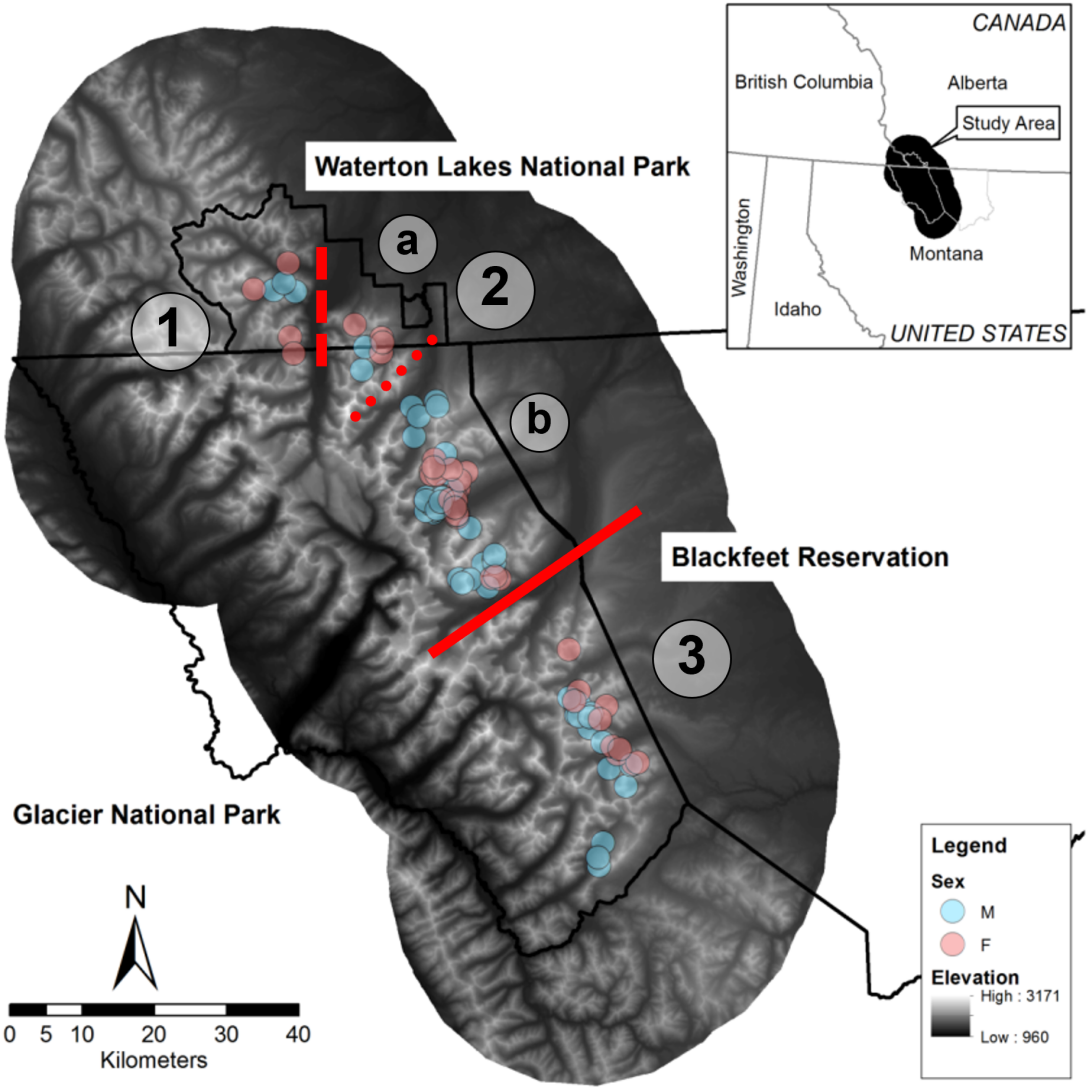
Map of the study area in the Waterton-Glacier International Peace Park and the Blackfeet Indian Reservation. Centroids of all GPS locations collared bighorn sheep (*Ovis canadensis*) during 2002 –2012 that were used to document contacts are shown (blue for males, pink for females). Red lines indicate possible breaks in the bighorn sheep population structure and line type indicates relative permeability of barriers (solid for no indirect contacts, dashed for few direct and indirect contacts, and dotted for no direct contacts but many indirect contacts). (1) West Waterton, (2) North Glacier (a: East Waterton, b: Many Glacier), (3) South Glacier.

We conducted this study in the Waterton-Glacier International Peace Park and the Blackfeet Indian Reservation (Fig. 1). This area comprises Glacier National Park in Montana, United States (∼400,000 ha), which is managed by the National Park Service, Waterton Lakes National Parks in Alberta, Canada (50,000 ha), which is managed by Parks Canada, and the western edge of the Blackfeet Indian Reservation (780,000 ha), which is managed by the Blackfeet Nation. Glaciations in the Pleistocene formed major topographical features, including many glacial lakes, moraines, and cirques, and left active glaciers (Thornberry, 2004). The Continental Divide of the Rocky Mountains runs north to south through the park, and greatly influences precipitation; elevations range from 960 to 3,171 m, and valleys east of the divide feature extensive open grassy slopes. Conifer forest (46.08%), grass (14.90%), scrub/shrub (14.85%), barren (7.92%), and deciduous forest (7.71%) are dominant land cover types in the overall study area, which we delineated based on a 25 km buffer around all bighorn GPS locations (Crown Managers Partnership, https://www.sciencebase.gov/catalog/item/51102e04e4b048b5cead853b).

Although no formal population estimates of bighorn in the park exist, bighorn are considered a species of management concern in Glacier National Park (MJ Biel, pers. obs., 2023), and historical counts suggest that ∼500 bighorn comprise this putative metapopulation (Flesch et al., 2022). No hunting occurs in the park, but the Blackfeet Nation manages a limited hunt for bighorn outside the park boundary, and a few poaching incidents occurred inside the park boundary during this study in the northern part of Glacier National Park. Mountain lions are likely the primary predator of bighorn adults in this area, but other predators such as grizzly bears (*Ursus arctos*), black bears (*U. americanus*), and eagles (*Aquila chrysaetos*) have been documented near kills in the park (MJ Biel, pers. obs., 2022) and gray wolves (*Canis lupus*), coyotes (*Canis latrans*), wolverines (*Gulo gulo*), are also present (Sawyer & Lindzey, 2002).

Prior research on pathogens of bighorn in the Waterton-Glacier International Peace Park identified two divisions in the bighorn population related to two natural geographic features. Specifically, De la Fuente et al. (2007) identified a division at the St. Mary Lake Valley by investigating exposure to *Anaplasma ovis*, and Ott et al. (2009) identified a division at the Belly River by investigating *Bibersteinia trehalosi* (formerly *Pasteurella trehalosi)* DNA sequences. Both pathogens were previously linked to respiratory disease in bighorn (Jaworski, Hunter & Ward, 1998; Rudolph et al., 2003; Weiser et al., 2003; Ott et al., 2009).

### Radiotelemetry

From November 2002 to April 2007 and March 2009 to April 2012, 97 bighorn (54 males, 43 females) were captured and fitted with global positioning system (GPS) collars (model 3500; Telonics, Inc., Mesa, Arizona, USA) using a combination of ground and aerial darting (Fig. S1). Collars were programed to record location data starting immediately after capture and to break away approximately 1 year after deployment. To address multiple objectives, collars were distributed across the entire bighorn range at the Waterton-Glacier International Peace Park. Due to logistical and funding constraints, such as restrictions on the use of helicopters to capture animals in a national park and limited accessibility to sites when they were covered in snow, data collection for this project spanned ∼10 years and only a limited number of collars were active at the same time. We estimated age based on horn annuli (Geist, 1966) and relatedness based on microsatellite genotypes from blood or hair samples (Buchanan et al., 1993; Forbes & Hogg, 1999). Microsatellite analysis revealed that three bighorn (two males, one female) had identical genotypes to other bighorn captured and collared in other years; we treated bighorn with identical microsatellite genotypes as the same individual. For the purposes of this study, we used GPS locations collected at 5-hour intervals, the longest programmed fix interval. All bighorn capture and handling protocols were approved by the US Geological Survey Institutional Animal Care and Use Committee (#20040413) and complied with US Geological Survey guidelines for animal care including measures to follow 3R tenets and bighorn-specific guidelines (Supplemental Text 1; Jessup, Mohr & Feldman, 1982; Kock et al., 1987; Kreeger, Raath & Arnemo, 2002).

### Data analysis

To understand contacts of bighorn, we conducted analyses focused on (1) where the divisions between subpopulations in the metapopulation are located based on indirect contacts, (2) how characteristics of individuals and dyads influenced direct contact frequency, (3) when direct contacts occurred, and (4) where direct contacts occurred on the landscape. We defined a direct contact as *simultaneous* GPS locations of two bighorn within a specified distance criterion and an indirect contact as GPS locations of two bighorn within a specified distance criterion regardless of timing. Various distance criteria have been used in other ungulate studies with GPS locations (Ramsey et al., 2002; Schauber, Storm & Nielsen, 2007; Kjær, Schauber & Nielsen, 2008; Schauber et al., 2015), and a distance of 25 m is comparable to GPS errors for the collars used in this study. Given this GPS error, the probability that a directly transmitted pathogen would spread between bighorn within this distance is higher because they are more likely to be in close physical proximity.

For analyses, we calculated variance inflation factors (VIF) and Pearson’s correlation coefficients (*r*) for all continuous explanatory variables. We excluded explanatory variables with VIF > 10 (Vittinghoff et al., 2012) and one of the pairs of variables with *r* ≥ —0.6— based on biological importance and interpretability. Using a multiple model theoretic approach to compare our hypotheses, we ranked models based on Akaike’s information criterion adjusted for small sample sizes (AICc; Burnham & Anderson, 2011). We share detailed results from models within 2 ΔAIC. All analyses were conducted in the R statistical computing environment (R Core Team, 2019), and all data visualizations were produced using the R package *ggplot2* (Wickham, 2016).

### Data censoring

To ensure independence of bighorn locations, movement, and associations, we identified bighorn that moved in a correlated fashion during any part of the year by calculating the dynamic interaction index (Long et al., 2014; Fig. S2) and excluded one of those individuals of the dyad at random from all further analyses. To account for the uneven distribution of collared bighorn across years during the study, we included a variable for the number of collared bighorn in the general area (within 400 m, the mean observed step length of a bighorn) in all models. We also standardized contacts by the number of days both collars in a dyad were active (*i.e.,* daily contact rate) to remove biases created by dyads active for a longer duration. All data with missing covariates were excluded from the analyses.

### Where are the divisions among subpopulations?

Because many bighorn were not simultaneously collared, we conservatively assessed connections among bighorn by classifying indirect contacts (*i.e.,* either at the same or separate time) using a contact distance criterion of 25 m. We constructed a network diagram where edges represent the number of indirect contacts between bighorn in the R package *igraph* (Csardi & Nepusz, 2006). We identified subpopulation partitions using the *group* function, which separates clusters of animals based on whether connections between 2 animals can be made through linkages with other animals. This method identifies areas in the network where there are no indirect contacts. We also focused on parts of the network that were only connected by one or two individuals. To further investigate whether any of these indirect contacts may have been direct interactions, we constructed a network diagram of contacts that occurred simultaneously (*i.e.,* direct contacts). We calculated direct contacts using the R package *spatsoc* (Robitaille, Webber & Van der Wal, 2019). We repeated the analysis with more relaxed contact distance criteria (50 and 100 m) to test the importance of the distance threshold. Based on population structure results, we conducted the remainder of the analyses with an indirect contact distance criteria of 25 m.

Additionally, we examined the indirect contact structure based on previously delineated social groups (K Keating, retired US Geological Survey, pers. comm. 2014). For this analysis, we used social groups as nodes and number of indirect contacts between social groups as edges. Using these social groups, we created a bipartite network separated by the social group sex and quantified the number of same sex groups and opposite sex groups with which each group came into indirect contact. We tested differences between proportion of indirect contacts made with opposite sex social groups (number of opposite sex groups/total number of groups contacted) and proportion of indirect contacts made with same sex social groups (number of same sex groups/total number of groups contacted) with a Wilcoxon signed rank test (*α* = 0.05).

### How do dyad characteristics influence contact rate?

To investigate which intrinsic variables were important to the strength of association between dyads (*i.e.,* frequency of direct contact), we calculated pairwise metrics for each dyad, considering volume of intersection (Millspaugh et al., 2004), a measure of joint space use; dynamic interaction index (Long et al., 2014), a measure of correlation in animal movements; and Queller and Goodnight estimator from microsatellite data (QGM; Queller & Goodnight, 1989), a measure of relatedness. For volume of intersection and dynamic interaction index, we calculated overall metrics in addition to seasonal metrics for winter (December–April), lambing (May–July), and fall (August–November). We categorized bighorn into two age classes (yearling or adult) and coded homophily of age class and sex as binary variables (1 for a match and 0 for a mismatch in age class or sex).

We used the multiple regression quadratic assignment procedures (MRQAP) with double-semi-partialing and 1,000 permutations (Dekker, Krackhardt & Snijders, 2007) to produce conservative correlation coefficient estimates and *p*-values through matrix permutations (Albery et al., 2021) using the R package *asnipe* (Farine, 2013). This method was developed specifically for network data to account for non-independence in dyad observations. We standardized contact values by dividing the frequency of contacts by the number of days both collars were active. To focus on neighboring bighorn, we only included dyads that had at least one simultaneous location within 100 m. We regressed the contact frequency against dyad characteristics (volume of intersection, dynamic interaction index, relatedness), homophily (sex, age class), and individual characteristics (sex, age in years) in univariate models and an additive global model.

### When do contacts occur?

To evaluate when contacts occurred, we fit a binomial generalized linear model (contact = 1) with a random effect for dyad and tested models that described time periods important to behavior including hour of day, time of day (*i.e.,* diurnal for 10:00 –16:00, crepuscular for 05:00 –9:00 and 17:00 –21:00, nocturnal for 22:00 –04:00), month, season (*i.e.,* winter, lambing, fall), and a behavior metric, speed from previous location (distance traveled/time difference). We conducted separate analyses for male-only, female-only, and male–female contacts because of known differences in life history characteristics.

### Where do contacts occur?

To evaluate the factors driving both overall bighorn locations and contact locations, we used a three-stage approach. First, we modeled general bighorn habitat use. Next, we modeled contact locations conditional on bighorn locations. Finally, we created a map of contact probability by combining habitat use models with contact models because a contact can only occur where bighorn are present. Again, we conducted separate analyses for male-only, female-only, and male–female contacts because of known differences in life history characteristics. We centered and scaled continuous explanatory variables (mean = 0 and SD =1) and considered linear, quadratic, and pseudo-threshold terms based on bighorn ecology (Table 1).

For the first stage, we modeled general bighorn habitat use using a step-selection function (*i.e.,* case-conditional approach; Fortin et al., 2005; Thurfjell, Ciuti & Boyce, 2014). We modeled habitat use because contacts are conditional on bighorn locations. For each used location, we identified five potential locations originating from the source point by randomly sampling from the distribution of step lengths and turn angles of other collared bighorn. In our analysis, we paired each used location with these five available locations and fit habitat use models using a conditional logistic regression in the package *mclogit* (Elff, 2018). We selected explanatory variables from the literature in three broad categories representing resources, survival, and disturbance (Table 1). We tested each variable and transformations of the variable to determine which term (linear, quadratic, or pseudo-threshold) was most appropriate. We preferentially selected linear forms of variables if the transformed terms performed similarly to the linear form to simplify interpretations. We then built a global model with those terms and applied that model to male-only, female-only, and male and female data. To understand differences in locations that males selected compared to locations females selected, we first classified probabilities of habitat use for each sex by decile (1 for the locations with the highest 10% probability of use values, 10 for locations with the lowest 10% probability of use values). We calculated Spearman’s rank correlation to assess the similarity of habitat use between the sexes.

For the second stage, we modeled contact locations relative to bighorn locations. To reduce zero inflation in our models (contacts not recorded because another collared bighorn was not nearby), we only included non-contact locations with simultaneous GPS locations within 500 m (3rd quartile of step length in 5 h of any bighorn). This allowed us to meet a reasonable assumption that a collared bighorn was available for contact given the distribution of step lengths of collared bighorn, the relatively large bighorn population size, and the small portion of bighorn collared in the population. To assist model convergence, we limited the analysis to dyads with five or more contacts. We fitted contact models using the *glmer* function in the *lme4* package (Bates et al., 2015) with a binomial distribution and logit link function. We included dyads as a random effect to account for varying number of contacts among dyads. We used the same global models from the habitat use models, so we could directly compare the coefficients of the explanatory variables. As in the habitat use analysis, we ranked probabilities of contact occurrences into deciles (1 for highest probability of contact, 10 for lowest probability of contact). To quantify how similar locations of contact were between male-male dyads, female–female dyads, and male–female dyads, we calculated Spearman’s rank correlations.

For the third stage of the analysis, we constructed a predictive map of contact locations using a joint distribution of general habitat use and contact locations given habitat use.

\begin{eqnarray*}Pr(Contact)=Pr \left( Habitat~use \right) \ast Pr(Contact{|}Habitat~use). \end{eqnarray*}

Finally, we assessed support for the post hoc hypothesis that contact probabilities given habitat use were higher in low to mid quality habitat. We calculated Spearman’s rank correlation to assess how similar the ranked probabilities of use and the ranked probabilities of contact were, comparing probabilities of female habitat use to female–female dyad contacts, male habitat use to male-male dyad contacts, and joint male and female habitat use to male–female dyad contacts.

## Results

During 2002–2012, we recorded a total of 168,380 GPS locations (n_M_ = 84,886, n_F_ = 83,494) from 97 bighorn (54 males, 43 females) (Table S1, Graves et al., 2023). We identified 138,469 possible indirect contacts using the 10 m distance threshold (n_F−F_ = 47,470, n_M−M_ = 40,943, n_M−F_ = 50,056) and 1,259,882 possible indirect contacts using the 25 m distance threshold (n_F−F_ = 443,426, n_M−M_ = 372,500, n_M−F_ = 443,956). We identified 5,324 direct contacts (n_F−F_ = 2,098, n_M−M_ = 2,716, n_M−F_ = 510) between 252 dyads and censored 30 dyads that were within the same group (16 female–female dyads with n_F−F_ = 870 contacts, 12 male-male dyads with n_M−M_ = 1,492 contacts, 2 male–female dyads with n_M−F_ = 2 contacts) as indicated by correlated movement during any part of the year. We analyzed 2,960 direct contacts (n_F−F_ = 1,228, n_M−M_ =1,224, n_M−F_ = 508) between 222 dyads among 558 possible dyads to explore how dyad characteristics influenced direct contact rates. After we removed locations with missing covariate data and censored dyads that had fewer than five direct contacts, we analyzed 2,091 direct contact locations (n_F−F_ = 956, n_M−M_ = 895, n_M−F_ = 240) between 109 dyads (37 female–female, 45 male-male, and 27 male–female dyads) to investigate where contacts occurred.

### Where are the divisions among subpopulations?

**Figure 2:**
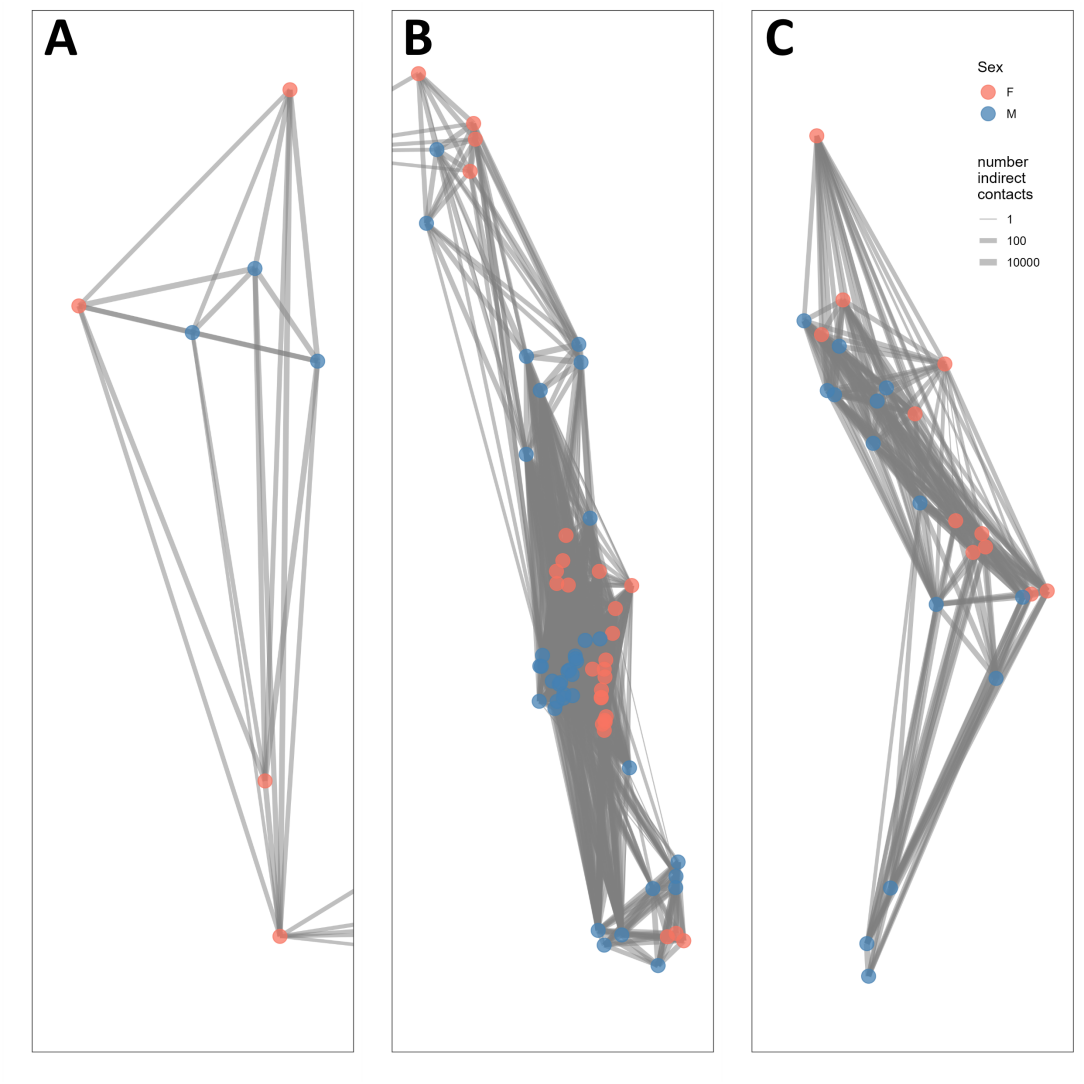
Network structure of bighorn sheep (*Ovis canadensis*) in Waterton-Glacier International Peace Park during 2002 –2012. Each panel represents a subpopulation based on the connections of indirect contacts. (A) West Waterton, (B) North Glacier, (C) South Glacier. Indirect contacts were defined as GPS locations of 2 bighorn sheep within 25 m regardless of timing. Each node represents the centroid of GPS locations of each collared bighorn sheep. Node color represents sex (pink for females, blue for males) and line width represents the number of indirect contacts. Lines extending to the right of panel A, from the lowest dot, are connected to lines extending to the left of panel B and represents a single individual from West Waterton subpopulation connected to multiple individuals in the North Glacier subpopulation. See Fig. 1 for overall configuration.

The indirect contact network structure revealed that this population of bighorn was structured into two groups separated by the St. Mary Lake Valley, a forested valley running east–west, and that the northern group could be split into two subgroups separated by Waterton Lake (Fig. 2). We refer to these three groups (from north to south) as the West Waterton subpopulation, the North Glacier subpopulation, and the South Glacier population.

The West Waterton subpopulation and the North Glacier subpopulation were connected by one female bighorn that made a long-distance movement from west of Waterton Lake to east of Waterton Lake and returned thereafter. We did not record any direct contacts between this female and any other collared bighorn using the shortest distance criterion of 25 m, but we recorded direct contacts with up to three other female bighorn in the east Waterton area using the longer distance criteria of 50 and 100 m. Direct contact network analysis also revealed that there may be another split within the North Glacier subpopulation across the Belly River, a relatively broad open valley.

When we compared indirect network structure with male and female social groups that were previously classified based on spatial use pattern, we found that greater proportions of indirect contacts occurred among opposite sex social groups than same sex social groups (*V* = 24, *P* = 0.041, Fig. S3). Specifically, male groups indirectly contacted more female groups than other male groups (*V* = 0, *P* = 0.035).

### How do dyad characteristics influence contact rate?

Strength of dyad associations were best predicted by dyad characteristics and homophily covariates, but there was no support for individual characteristics (*i.e.,* age or sex; Table 2). Specifically, bighorn with higher home range overlap during the summer had higher levels of associations (*β*_summerV I_ = 0.116, *P* = <0.001). Same-sex dyads had higher levels of association (*β*_homophily_sex_ = 0.022, *P* = <0.001): female–female dyads had the strongest associations (*β*_FF_ = 0.020, *P* = <0.001) followed by male-male associations (*β*_MM_ = 0.015, *P* = 0.005).

**Table 2:** Effects of dyad characteristics on contact rates of bighorn sheep in the Waterton-Glacier International Peace Park in Montana, USA and Alberta, Canada and Blackfeet Indian Reservation during 2002–2012. Models were run in R package asnipe using multiple regression quadratic assignment procedures (MRQAP) with double Dekker semi-partialing and 1,000 permutations.

Model	Covariate	Coefficient	*P*-value
Dyad_sex_ + VI_summer_ + Relatedness^2^	Intercept	−0.012	0.976
	Female–female dyad	0.020	<0.001
	Male-male dyad	0.015	0.005
	VI_summer_	0.116	<0.001
	Relatedness	0.012	0.232
	Relatedness^2^	−0.104	0.913
Homophily_sex_ + Homophily_age_class_	Intercept	0.002	0.395
	Homophily_sex_	0.022	<0.001
	Homophily_age_class_	0.006	0.296
Individual_sex_ + Individual_age_	Intercept	0.011	0.058
	Individual_sex(M)_	0.002	0.325
	Individual_age_	0.001	0.119

**Notes.**

* Volume of intersection (VI) by season.

### When do contacts occur?

**Figure 3:**
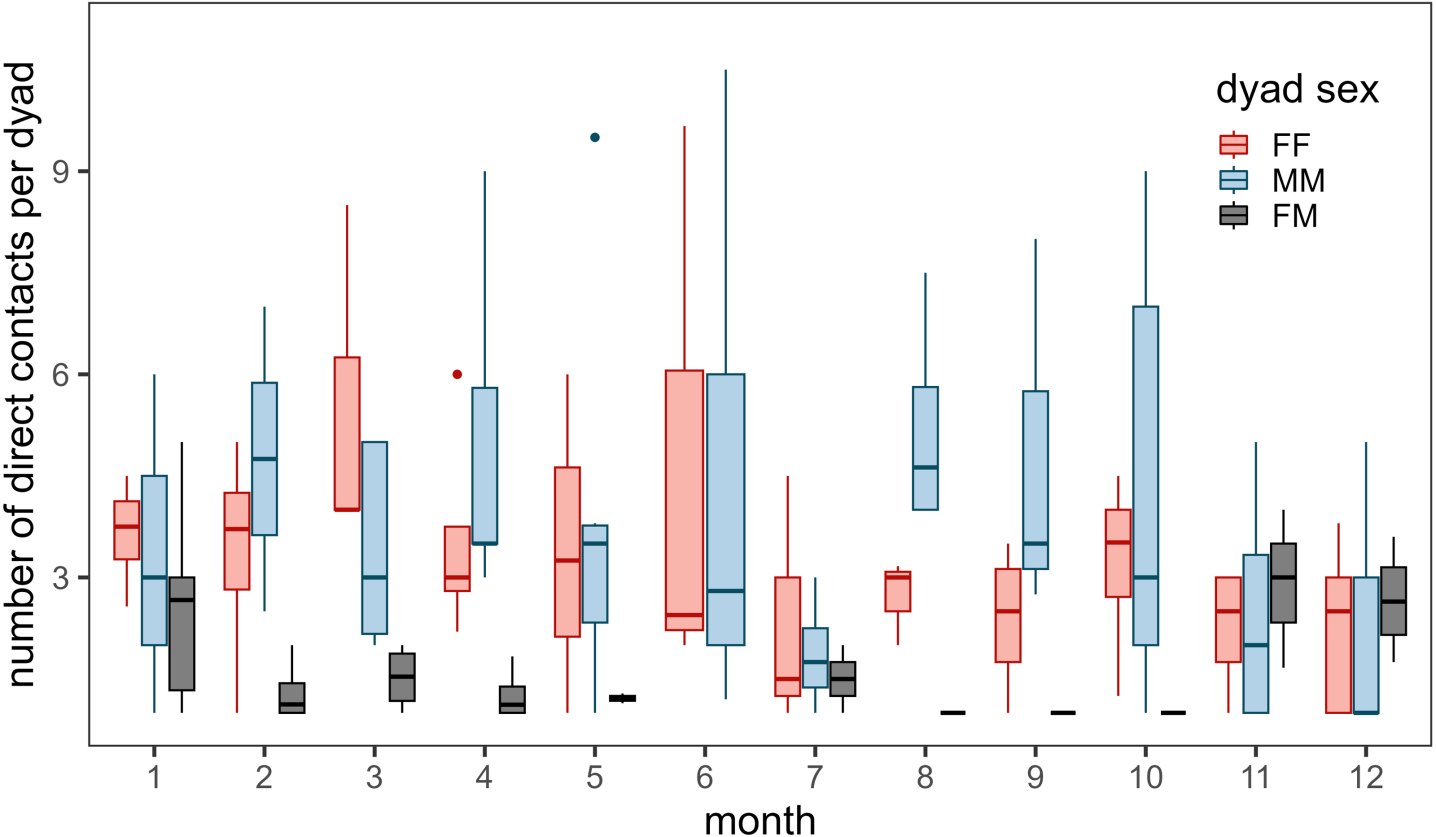
Annual number of direct contacts per dyad by month during 2002–2012. Direct contacts were defined as simultaneous GPS locations of two bighorn sheep (*Ovis canadensis*) ≤ 25 m. Colors represent dyad type (pink for female–female dyads, blue for male-male dyads, and black for male–female dyads). Only dyads with at least 1 direct contact during the month were included. Box and whisker plot depicts median as middle line, inter-quartile range as box, 1.5 times the inter-quartile range as whiskers, and outliers as dots. Note: number of direct contacts should not be compared between dyad types given uneven collar deployments throughout the study.

Contacts occurred most frequently during March for female–female dyads, during August for male-male dyads, and during November, December, and January for male–female dyads (Fig. 3). Few contacts occurred between female–female dyads in July and male–female dyads during the summer (June - October). We detected more contacts when more collared bighorn occurred in the general area (*β*_bighorn,MM_ = 0.81 ± 0.07, *β*_bighorn,FF_ = 0.66 ± 0.06, *β*_bighorn,MF_ = 0.69 ± 0.08) and when bighorn moved more slowly (*β*_speed,MM_ = −0.14 ± 0.05, *β*_speed,FF_ = 0.07 ± 0.03, *β*_speed,MF_ = −0.23 ± 0.08; Fig. S4). Contacts also occurred more frequently at night than during crepuscular and daylight hours for female–female and male–female dyads.

### Where do contacts occur?

#### General habitat use

Bighorn used similar areas regardless of sex (male *vs.* female step-selection function rank: r_s_ = 0.98, *p* < 0.0001). General use habitat models only included resource and survival variables (Table 3, Tosa & Graves, 2023). The three disturbance variables (distance to helicopter route [d.heli], distance to road [d.road], and distance to trail [d.trail]) that we tested had opposite effects on resource selection than expected in univariate models and thus were not included in the global models (Fig. S5). Slope was correlated with distance to escape terrain and was therefore excluded from the global model. Relative to available locations, bighorn used areas with greater normalized difference vegetation index (NDVI), instantaneous rate of green-up (IRG), and solar radiation index (SRI), areas of intermediate elevation and distance to perennial streams, and less rugged areas with low canopy cover and low snow water equivalent that were close to escape terrain and close to mineral licks (Figs. S6 and S7).

**Table 3:** Coefficients of step selection model of bighorn sheep habitat in the Waterton-Glacier International Peace Park and the Blackfeet Indian Reservation during 2002 –2012.

	**Female-only**			**Male-only**			**All**		
	**Linear**	**Quadratic**	**Pseudo-** **threshold**	**Linear**	**Quadratic**	**Pseudo-threshold**	**Linear**	**Quadratic**	**Pseudo-threshold**
**Resource**									
SWE	−0.05 ± 0.01			−0.05 ± 0.01			−0.05 ± 0.01		
SRI	0.12 ± 0.01			0.16 ± 0.01			0.14 ± 0.01		
NDVI	0.11 ± 0.02			0.18 ± 0.01			0.15 ± 0.01		
IRG	−0.09 ± 0.02	0.13 ± 0.01		0.001 ± 0.02	0.10 ± 0.01		−0.04 ± 0.01	0.11 ± 0.01	
Elevation	−1.13 ± 0.11		1.17 ± 0.11	−2.41 ± 0.12		2.37 ± 0.12	−1.70 ± 0.08		1.70 ± 0.08
d.mlick	−0.24 ± 0.05		−0.19 ± 0.02	−0.20 ± 0.04		−0.12 ± 0.01	−0.21 ± 0.03		−0.15 ± 0.01
d.h2o.per	0.10 ± 0.01	−0.10 ± 0.01		0.14 ± 0.01	−0.04 ± 0.01		0.13 ± 0.01	−0.06 ± 0.01	
**Survival**									
VRM	−0.02 ± 0.01		−0.01 ± 0.01	−0.02 ± 0.01		−0.04 ± 0.01	−0.02 ± 0.01		−0.02 ± 0.01
d.esc	−0.40 ± 0.01			−0.44 ± 0.01			−0.42 ± 0.01		
Canopy	−0.29 ± 0.01			−0.24 ± 0.01			−0.26 ± 0.01		

**Notes.**

Variable abbreviations SWE snow water equivalent SRI solar radiation index NDVI normalized difference vegetation index IRG instantaneous rate of green-up d.mlick distance to mineral lick d.h2o.per distance to perennial water source VRM vector ruggedness metric d.esc distance to escape terrain canopy canopy cover

#### Contact locations given habitat use

As we predicted, contacts were most likely to occur at similarly ranked locations across all dyad types (male-only *vs.* female-only dyad contacts: r_s_ = 0.66, *p* < 0.0001; male-only *vs.* male–female dyad contacts: r_s_ = 0.55, *p* < 0.0001; female-only *vs* male–female dyad contacts: r_s_ = 0.88, *p* < 0.0001, Table 4; Fig. S9, Tosa & Graves, 2023). For all types of dyads, contacts occurred most at intermediate distances from perennial streams and intermediate IRG. Contacts between female–female dyads occurred in areas close to mineral licks, in locations with high SRI, high snow water equivalent, high canopy cover, and high ruggedness. Contacts between male-male dyads occurred in areas close to mineral licks, low canopy cover, low ruggedness, and far from escape terrain. Contacts between male–female dyads occurred at intermediate distances from mineral licks, at low elevations, and at locations with high NDVI (Table 4; Fig. S10).

**Table 4:** Coefficients of the resource selection model of bighorn sheep contacts in the Waterton-Glacier International Peace Park and the Blackfeet Indian Reservation during 2002–2012.

	**Female-Female**			**Male-Male**			**Male-Female**		
	**Linear**	**Quadratic**	**Pseudo-** **threshold**	**Linear**	**Quadratic**	**Pseudo-** **threshold**	**Linear**	**Quadratic**	**Pseudo-** **threshold**
Intercept	−1.72 ± 0.06			−1.72 ± 0.06			−1.91 ± 0.15		
**Resource**									
SWE	0.34 ± 0.06			−0.05 ± 0.06			−0.14 ± 0.14		
SRI	0.15 ± 0.05			−0.05 ± 0.05			−0.005 ± 0.10		
NDVI	0.06 ± 0.05			−0.04 ± 0.05			0.26 ± 0.09		
IRG	0.35 ± 0.18	−0.47 ± 0.17		0.47 ± 0.18	−0.44 ± 0.18		1.27 ± 0.37	−1.61 ± 0.40	
Elevation	0.79 ± 0.65		−0.73 ± 0.65	−0.87 ± 0.62		0.86 ± 0.62	6.01 ± 1.32		−6.88 ± 1.33
d.mlick	−0.15 ± 0.14		−0.18 ± 0.12	0.03 ± 0.06		−0.09 ± 0.04	−1.22 ± 0.32		1.68 ± 0.34
d.h2o.per	0.41 ± 0.17	−0.30 ± 0.16		0.31 ± 0.17	−0.39 ± 0.17		0.52 ± 0.34	−0.26 ± 0.32	
**Survival**									
VRM	0.15 ± 0.07		0.02 ± 0.07	−0.08 ± 0.07		0.08 ± 0.07	−0.15 ± 0.15		0.42 ± 0.16
d.esc	0.03 ± 0.04			0.16 ± 0.04			0.11 ± 0.09		
Canopy	0.08 ± 0.04			−0.22 ± 0.06			−0.03 ± 0.08		

**Notes.**

Variable abbreviations SWE snow water equivalent SRI solar radiation index NDVI normalized difference vegetation index IRG instantaneous rate of green-up d.mlick distance to mineral lick d.h2o.per distance to perennial water source VRM vector ruggedness metric d.esc distance to escape terrain canopy canopy cover

### Contact locations *vs.* general habitat use

**Figure 4:**
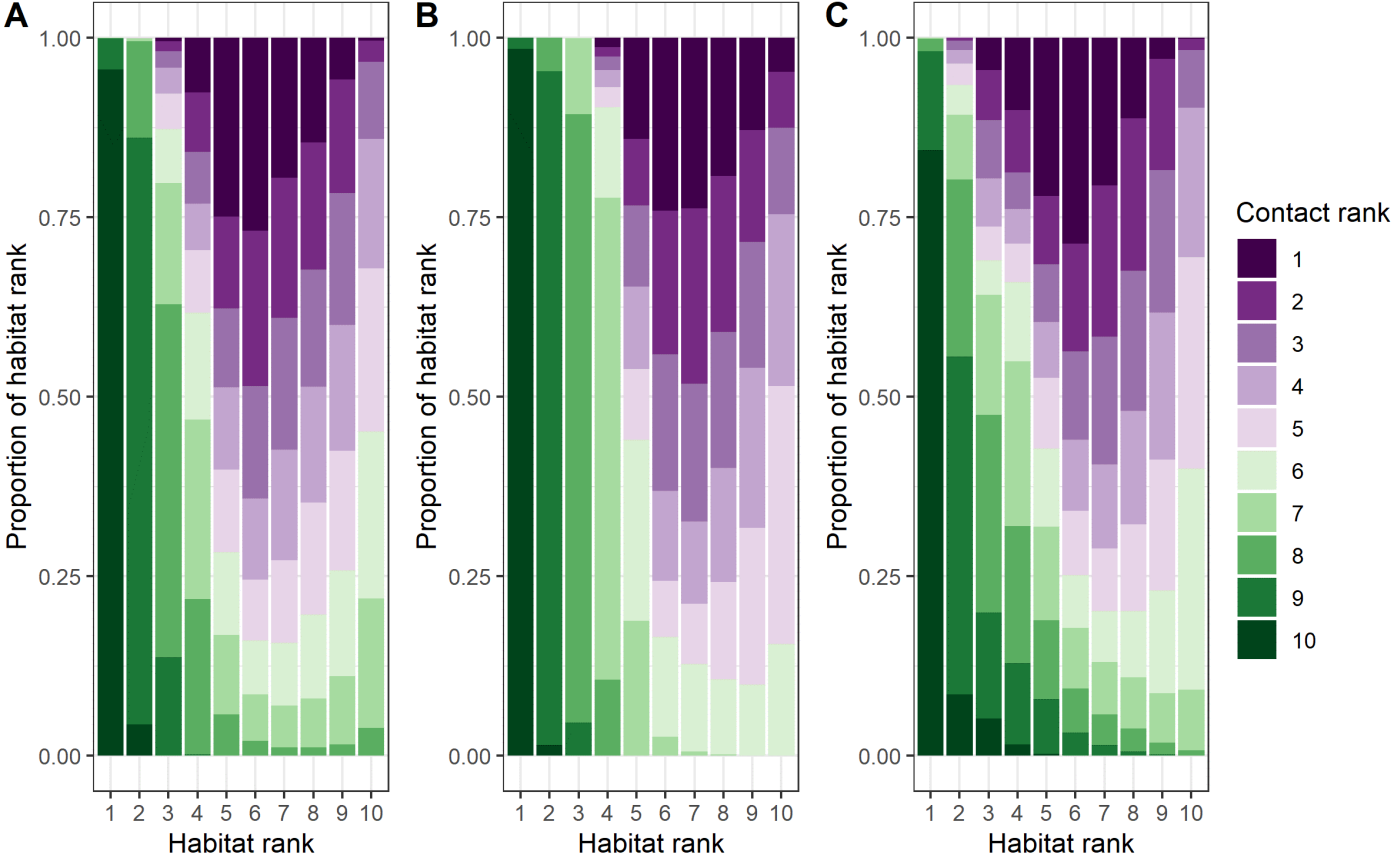
Comparison of ranked probability of general use and ranked probability of direct contact given general use by bighorn sheep (*Ovis canadensis*) in the Waterton-Glacier International Peace Park. Probability of general use and probability of direct contact given general use ranked by decile (1 = least probable, 10 = most probable). Colors represent ranked probability of direct contact given general use. (A) female-only, (B) male-only, and (C) male and female dyads of bighorn sheep.

Female–female, male-male, and male–female dyads contact location ranks were inversely related to habitat use location ranks (female-only dyads: r_s_ = −0.61, *p* < 0.0001; male-only dyads: r_s_ = −0.76, *p* < 0.0001; male–female dyads: r_s_ = −0.56, *p* < 0.0001; Fig. 4 and Fig. S9, Tosa & Graves, 2023). The most pronounced differences between contact locations and habitat locations across dyad types related to survival variables: canopy cover, distance to escape terrain, and ruggedness (Figs. S7 and S10). Differences in female–female contact and habitat use probabilities were also driven by opposite responses to snow water equivalent (SWE): female bighorn generally selected against areas with high SWE, but when they were in areas with high SWE, they were more likely to contact other female bighorn (Table 1, Figs. S7 and S10). Interestingly, male–female dyads were less likely to contact each other close to mineral licks, even though both sexes generally selected to be closer to mineral licks.

## Discussion

Our results show that contact analysis is a valuable method for understanding animal behavior and fitness tradeoffs of sociality. With close proximity to conspecifics, bighorn appear to gain an advantage in a challenging environment that they would not have if they were solitary, despite risks of resource competition and disease. Although spatial proximity does not necessarily equate to physical contact between two individuals (Tosa, Schauber & Nielsen, 2015) and rates of disease transmission may vary by individual or dyad characteristics (Manlove et al., 2017), we demonstrate that the distance criteria we used appears to serve as an appropriate surrogate for direct physical contacts that are necessary for disease transmission given the consistency between breaks in the population identified through the indirect contact network and divisions in previous disease status.

At the landscape scale, we demonstrated that mapping indirect and direct contact network structure can reveal information similar to that gained through disease exposure and genetic analyses. Although we had a limited number of collars deployed each year (maximum of 13), we identified 1 distinct barrier (St Mary’s Lake Valley) and 2 other partial barriers (Belly River and Upper Waterton Lake) that naturally separate 3–4 subpopulations of bighorn in this system (Fig. 1). Two of these possible barriers (St. Mary’s Lake Valley and the Belly River) were consistent with divisions identified by other methods that focused on exposure to diseases such as *Anaplasma ovis* and *Bibersteinia trehalosi* that require direct contact (De la Fuente et al., 2007; Ott et al., 2009). Moreover, analyses on the genomic structure of this population confirmed limited long-term movement across the St. Mary Lake Valley (Flesch et al., 2020; Graves & Flesch, 2020). As spatial proximity is a necessary component of the mechanisms for genetic mixing and disease transmission, this demonstrates contact analyses reflect the most frequent mixing. Consequently, these three possible barriers that we identified represent important locations for disease management, should respiratory disease infect any section of the population (Manlove et al., 2014).

Connections between the east and west Waterton subpopulations were surprising, given the connections were formed by female–female dyads. This was unexpected because males typically have larger home ranges, have been observed making long distance movements during the rut, and therefore are more likely to contact other bighorn (Hogg, 2000; DeCesare & Pletscher, 2006). We also found that social groups of bighorn were more likely to contact social groups of the opposite sex, which highlights the importance of mating season for disease transmission (Fig. S3). Because contacts between the West Waterton and East Waterton subgroups occurred within 4 days (25 –29 July 2006), this may represent a single exploratory movement by the female from the West Waterton subgroup to the east pre- or post-dispersal. Limited research currently informs the drivers and frequencies of these rare long-distance movements although they have consequences to disease and genetic mixing.

Concerning factors that influenced the associations between dyads of bighorn, female–female dyads with greater space-use overlap during the summer season had the greatest probability of contact (Table 2). Relatedness was not a significant indicator of contact, suggesting that kinship was not necessary for associations between individuals from separate social groups and that sociality may be beneficial for survival of the individual (direct fitness) instead of increased fitness through related family members (indirect fitness) (Hamilton, 1964). This is consistent with bighorn in Alberta, Canada, where kinship appeared to play a limited role in the social system (Festa-Bianchet, 1991). The other factors, homophily of sex and space-use overlap, indicated that bighorn sociality is structured based on sex and proximity during the summer as described by Geist (1971) and others (Festa-Bianchet, 1991; Ruckstuhl, 1998).

Regarding the effect of landscape and vegetation variables, we found that bighorn habitat use was highly influenced by survival variables and less so by resource variables. Similar to other studies, this suggests that predation has strong selective pressures on locations of bighorn and provides evidence that bighorn operate in a “landscape of fear” (Laundré, Hernández & Altendorf, 2001). The disturbance variables that we explored had effects opposite to what we expected on bighorn space use (*i.e.,* bighorn selected for disturbance variables instead of selecting against disturbance variables). This may reflect the placement of trails, roads, and helicopter routes in less steep areas that facilitate movement for both humans and bighorn, that provide visitors with opportunities to view wildlife and to recreate, or poor alignment of the map of helicopter routes with actual routes. Alternatively, the relative effects of these disturbances could be minimal given the levels of disturbance while bighorn were collared compared to survival and resource variables or bighorn may be habituated to anthropogenic disturbances inside this highly protected area.

The predicted probabilities of contact given habitat use varied greatly from the habitat use models and suggest bighorn are responding to the tradeoffs of being social (Fig. 4). For example, contact probabilities given habitat use were lower when nutritional quality of forage, indicated by IRG, was high (Fig. S10), which suggests that the benefits of social behavior are outweighed by competition for valuable resources. On the other hand, when resources were scarce (*e.g.*, low solar radiation index for male-male and male–female dyads or high snow water equivalent for female–female dyads), contact probabilities were higher, which suggests that the benefits of social behavior (*i.e.,* increased thermoregulation efficiency) outweighed the disadvantage of sharing other resources (Arnold, 1990). The benefits of sociality were more pronounced in the predicted probabilities of contact for survival variables than for the resource variables. In areas where bighorn were vulnerable to predation, contacts were more likely. For example, contacts between female–female dyads were more likely to occur where canopy cover was higher, and contacts between all dyad types were more likely to occur when bighorn were farther from escape terrain (Fig. S10). This supports hypotheses for the evolution of sociality based on anti-predator benefits such as the many eyes hypothesis (Lima, 1995; Rieucau & Martin, 2008), whereby animals can increase the probability of detecting a predator, and the dilution of risk hypothesis (Pulliam, 1973; Foster & Treherne, 1981), whereby animals decrease their risk of predation by splitting the risk between the animals in the social group. Alternatively, these areas of low-ranking habitat may coincide with the edges of social groups, where multiple social groups are most likely to overlap.

The distance to mineral licks variable reveals interesting behavior of bighorn (Fig. S6). Although mineral licks were important to both male and female bighorn as indicated through the habitat use analysis, the contact analysis reveals that the two sexes rarely use this resource at the same time (Figs. S7 and S10). This is consistent with observations of other mountain ungulates such as mountain goats (*Oreamnos americanus*) where males generally use mineral licks earlier in the year followed by females and family groups starting in June (Poole, Bachmann & Teske, 2010). The importance of mineral licks for male and female habitat use and for female–female and male-male contacts identifies valuable areas for potential bighorn population monitoring. There are only a handful of known mineral licks in the Glacier-Waterton International Peace Park (MJ Biel, pers. obs., 2022); therefore, this naturally limited resource creates ideal locations for monitoring populations of bighorn and other mountain ungulates. Mineral licks provide mountain ungulates access to elements including sodium, calcium, potassium, and magnesium that can be necessary to regulate body fluids such as blood (Hebert & Cowan, 1971), mediate the effects of secondary compounds in forage (Ayotte et al., 2006; Ayotte, Parker & Gillingham, 2008), and meet the physiological demands of lactation and other functions critical to the health of populations (Ayotte et al., 2006). As such, many animals make repeated visits to these mineral licks over their lifetime and often travel long distances (Rice, 2010). These areas, however, may also increase their risk of predation (Côté & Beaudoin, 1997; Sarmento & Berger, 2017) and increase rates of pathogen transmission through direct and indirect contact. Fomites, artificial and supplemental feeding sites, and watering holes that aggregate wildlife in concentrated areas can increase disease transmission rates in other systems (Cross et al., 2010; Paull et al., 2012; Sorensen, Van Beest & Brook, 2014).

Much of our analyses support previous findings concerning bighorn social systems and social structure. Analyses of the correlation of movement between bighorn revealed that bighorn behave in a fission–fusion social system. Dyads moved together during winter and spring but split apart during the summer (Fig. S2). We also documented variability in contact rates by dyad type and seasonality in the timing of contacts (Fig. 3). Female–female contacts were highest in March when vegetation starts to green-up and food resources are spatially restricted (Fig. S11). Female-male contacts were highest during the mating season through early winter (November–January), which corresponds to times of the year when movement probabilities of males were greatest (Borg et al., 2017). In contrast, male–female contacts were lowest during the summer when females separate from males to raise offspring (lambs). During the winter, when food resources are scarce, low ambient temperatures may force bighorn to huddle together for thermoregulation. Moreover, our analyses revealed that contacts were most likely when bighorn were moving slowly. This suggests that contacts occur during resting and foraging bouts instead of during large, directed movements in unfamiliar areas (*e.g.*, at a mineral lick *vs.* movements toward a mineral lick).

## Conclusion

Our study assesses social behavior of a wild, native, and large population of bighorn at the population, group, dyad, and individual scales. Using this unique 10-year GPS dataset of both male and female bighorn, we assessed social behavior among three dyad types–male-only, female-only, and male–female dyads–and modeled when, where, and with whom bighorn interact at close distances. Patterns of association were influenced by intrinsic dyad characteristics and by extrinsic characteristics such as the instantaneous rate of green-up, snow water equivalent, terrain ruggedness, and distance to escape terrain, representing resource availability and predation risk: same sex dyads had higher contact frequencies and bighorn in areas with lower resource availability and higher predation risk had higher conditional probabilities of contact.

Although we documented factors that influenced where and when contacts occurred between adult bighorn in this study, the initial purpose of this study was to create a bighorn habitat use map. Due to logistical constraints, all animal captures were conducted with ground-based darting, and it is likely that the bighorn represented in this study are biased toward those that use areas that are accessible to humans. During this study, the number of direct contacts may have been overestimated because we used a distance criterion as a proxy for direct contact. In other words, it is possible that individuals were within 25 m, but did not get close enough for transmission of respiratory disease (Tosa, Schauber & Nielsen, 2015). Given the large bighorn metapopulation and large spatial extent of the study, however, it is more reasonable to assume that the number and locations of direct contacts between bighorn were underestimated. Although we were unable to incorporate disease status of individuals and the effects that disease status might have on habitat use or dominance position (Edmunds et al., 2018) during this study, this study provides a valuable starting point to consider the connections between the selective pressures of disease, predation, and competition. Future studies could investigate changes in bighorn space use due to disease status to explore these relationships further.

Another consideration for this study is that much of the landscape is dynamic in this area. As snowpack, conifer encroachment, vegetation composition, and forage phenology change, the when and where of contacts could likewise change (Berman et al., 2020). This region is also susceptible to wildfires, and climate change models predict that wildfires will become more frequent and more severe (Barbero et al., 2015; Westerling, 2016). Most recently, the Reynolds wildfire at the St. Mary Lake division has significantly changed the vegetation in this area. As such, the divisions that we identified may no longer consist of the same barriers and populations may become more connected. Given the status of this metapopulation as one of only two large metapopulations in Montana, further research could evaluate changes in contact rates, especially across the St. Mary Lake barrier. This could help inform managers of management options should respiratory disease enter one of these subpopulations. Overall, our findings suggest that contact analyses that incorporate information about individuals and space can be a valuable method for understanding fitness tradeoffs of sociality and information on the who, when, and where of bighorn disease transmission potential at population, group, dyad, and individual scales.

##  Supplemental Information

10.7717/peerj.15625/supp-1 Supplemental Information 1 R scripts

10.7717/peerj.15625/supp-2 Supplemental Information 2 Bighorn GPS locations_do not publish

10.7717/peerj.15625/supp-3 Supplemental Information 3 Contact RSF Data_do not publish

10.7717/peerj.15625/supp-4 Supplemental Information 4 Direct contact data_do not publish

10.7717/peerj.15625/supp-5 Supplemental Information 5 Dyad structure_do not publish

10.7717/peerj.15625/supp-6 Supplemental Information 6 Supplemental Figures

10.7717/peerj.15625/supp-7 Supplemental Information 7 ARRIVE checklist

## Additional Information and Declarations

### Data Availability

The following information was supplied regarding data availability: The data is available at USGS: Graves, T.A., Tosa, M.I., Flesch, E.P., and Keating, K.A., 2023, Glacier Waterton International Peace Park bighorn sheep (*Ovis canadensis*), 2002-2012: U.S. Geological Survey data release, https://doi.org/10.5066/P9ANTBOF.
