# Supplementary material for: Bighorn sheep associations: understanding tradeoffs of sociality and implications for disease transmission
Source: PeerJ. 2023 Aug 8;11:e15625. doi: 10.7717/peerj.15625 (PMC10416771; doi:10.7717/peerj.15625)
Supplement: Supplemental Information 6 [file peerj-11-15625-s006.docx]

**Supplemental information to article: Bighorn sheep associations: understanding tradeoffs of sociality and implications for disease transmission**

Marie I. Tosa^1^, Mark J. Biel^2^_,_ Tabitha A. Graves^1^

^1^U.S. Geological Survey, Northern Rocky Mountain Science Center, 38 Mather Drive, PO Box 169, West Glacier, Montana, USA 59936

^2^Glacier National Park, National Park Service, West Glacier, Montana, USA 95536

**Supplemental Text 1. Details on IACUC protocols and handling outcomes**

**Table S1.** Attributes of bighorn sheep (*Ovis canadensis*) collared in the Waterton-Glacier International Peace Park during 2002-2012.

**Figure S1.** Collared bighorn sheep (*Ovis canadensis*) in the Waterton-Glacier International Peace Park during 2002-2012. Duration of active collars in (A) West Waterton Lakes National Park, (B) East Waterton Lakes National Park, (C) South Glacier area, and (D) North Glacier area. (E) Distribution of GPS locations across the study area

**Figure S2.** Correlation of movements of bighorn sheep dyads according to space-use overlap (volume of intersection) by sex. A) Female-female dyads, B) male-male dyads, and C) male-female dyads. Grey lines indicate the same dyad in different seasons. Lighter colors indicate yearling dyads, darker colors indicate adult dyads. Different line types used to help distinguish which points belong to the same dyad.

**Figure S3.** (A) Indirect contact network structure of bighorn sheep (*Ovis canadensis*) social groups (male = square, female = circle) and (B) comparison of indirect contact among social groups of the same sex and opposite sex in Waterton-Glacier International Peace Park during 2002 – 2012. Indirect contacts defined as GPS locations < 25 m apart. Social groups are named after local landmarks (starting with the leftmost group, females then males): GM = Goat Mountain, A = Ahern, IP = Iceberg-Ptarmigan, SS = Sofa-Sarcee, MD = Dungarvan, MB = Blakiston, VP = Vimy Peak, RC = Richards, SP = Scenic Point, DF = Dry Fork, SM = St. Mary, CM = Chief Mountain, MG = Many Glacier, WW = West Waterton, EW = East Waterton, STM = South Two Medicine, and NTM = North Two Medicine.

**Figure S4.** Variables from best model describing the timing of when contacts occurred between male-only (blue), female-only (pink), and male-female (black) bighorn sheep (*Ovis canadensis*) dyads in the Waterton-Glacier International Peace Park during 2002 – 2012. Note that time of day and season were categorical variables. Crepuscular hours were the reference category for nocturnal and diurnal categories and the fall season was the reference category for summer and winter.

**Figure S5.** Predicted probabilities of use from univariate models of a step-selection function for male and female bighorn sheep (*Ovis canadensis*) in the Waterton-Glacier International Peace Park during 2002 – 2012.

**Figure S6.** A) Global habitat selection models for males (blue), females (pink), and male and female (black) bighorn sheep (*Ovis canadensis*) and B) global contact models for male-only (blue), female-only (pink), and male-female (black) dyads of bighorn sheep in the Waterton-Glacier International Peace Park during 2002 – 2012. Variables are grouped by category: resource variables in top section, survival variables in the bottom section. Error bars represent standard errors. Shapes represent transformations to variables: pseudo-threshold (p; diamond), quadratic (q; circle), and centered and scaled (s; triangle).

**Figure S7.** Predicted probabilities of use for global models of discrete-choice step-selection functions for male-only (blue), female-only (pink), and male and female (black) bighorn sheep (*Ovis canadensis*) in the Waterton-Glacier International Peace Park during 2002 – 2012.

**Figure S8.** Predicted overall resource selection (1 = least likely to be used, 10 = most likely to be used), modeled as discrete-choice step-selection model. Habitat use of (A) female-only, (B) male-only, and (C) male and female bighorn sheep (*Ovis canadensis*) in the Waterton-Glacier International Peace Park during 2002 – 2012.

**Figure S9.**  Predicted resource selection of contacts, modeled as a resource selection function model (1 = least likely to contact, 10 = most likely to contact). Contacts between (A) female-female, (B) male-male, and (C) male-female dyads of bighorn sheep (*Ovis canadensis*) in the Waterton-Glacier International Peace Park during 2002 – 2012.

**Figure S10.**  Predicted probabilities of use from global models for contact resource selection functions for male-only (blue), female-only (pink), and male-female (black) dyads of bighorn sheep (*Ovis canadensis*) in the Waterton-Glacier International Peace Park during 2002 – 2012.

**Figure S11.** Day of year (DOY) vs. instantaneous rate of green up (IRG) of direct contact locations and locations where bighorn sheep (*Ovis canadensis*) where available for direct contact.

**Supplemental Text 1. Details on IACUC protocols and handling outcomes**

Steps in protocols to reduce pain, suffering, and distress included: the use of drugs with short induction times and antagonists, vital signs monitoring, reversal protocols if temperature exceeded 103.5 degrees and respiratory rate < 6 breaths/minute, administration of long-acting oxytetracyclien and vitamin-E selenium, disinfection at blood sample, dart location, and PIT tag sites, and the use of collars that had UV-degrading spacers and automatic releases.

The project had a lower capture mortality rate than expected, with 1 of 110 capture events leading to death. The death occurred ~73 hours post-handling in the 2nd animal handled for the project (1st day of handling). The necropsy determined the proximal cause of death was a fall, but several potential paths could have tied capture to the fall so protocols were adapted to include a higher dosage for antagonist to reduce likelihood of re-narcotization. Several other expected issues with handling occurred that were in the original protocols. Early reversals with the antagonists occurred 4 times: the dart hit a vein in 2 of these cases, causing heavy bleeding which was addressed with a hemostat. Post-handling observations (15 minutes, 2 hours= maximum possible) indicated alert/typical behavior. The other 2 cases were bighorns in poor condition that reached high temperature thresholds for reversal. Minor injuries in 3 other sheep. No abnormalities were observed in movement patterns for any of these 7 bighorns. This study uses archived data and humane endpoints were not required and were not explicitly stated in the IACUC protocols, but handling protocols that could have contributed to the one capture mortality were rapidly evaluated and addressed. Animals were re-sighted when possible and evaluated for signs of disease and injury.

**Table S1.** Attributes of bighorn sheep (*Ovis canadensis*) collared in the Waterton-Glacier International Peace Park during 2002-2012.

| **ANIMAL ID** | **GROUP** | **SEX** | **AGE** |
| --- | --- | --- | --- |
| 02-01 | MG | M | 8.5 |
| 02-03 | MG | M | 2.5 |
| 02-04 | A | F | 4.5 |
| 02-05 | A | F | 3.5 |
| 02-06 | A | F | 4.5 |
| 02-07 | A | F | 2.5 |
| 02-08 | A | F | 1.5 |
| 02-09 | MG | M | 1.5 |
| 03-01 | MG | M | 3.5 |
| 03-02 | MG | M | 4.5 |
| 03-03 | MG | M | 6.5 |
| 03-04 | A | F | 1.5 |
| 03-05 | MG | M | 11.5 |
| 03-06 | MG | M | 2.5 |
| 03-07 | A | F | 1.5 |
| 03-10 | SM | M | 4.5 |
| 03-11 | SM | M | 3.5 |
| 03-12 | SP | F | 3.5 |
| 03-13 | STM | M | 3.5 |
| 03-14 | DF | F | 3.5 |
| 03-15 | DF | F | 4.5 |
| 03-16 | A | F | 4.5 |
| 03-17 | SM | M | 4.5 |
| 03-18 | SM | M | 5.5 |
| 03-19 | A | F | 10.5 |
| 03-20 | A | F | 9.5 |
| 03-21 | MG | M | 10.5 |
| 03-22 | A | F | 6.5 |
| 03-24 | A | F | 5.5 |
| 04-01 | MG | M | 9.5 |
| 04-02 | MG | M | 4.5 |
| 04-03 | MG | M | 8.5 |
| 04-04 | MG | M | 3.5 |
| 04-05 | MG | M | 2.5 |
| 04-06 | NTM | M | 4.5 |
| 04-07 | NTM | M | 10.5 |
| 04-08 | STM | M | 6.5 |
| 04-09 | STM | M | 8.5 |
| 04-10 | MG | M | 4 |
| 04-11 | IP | F | 6 |
| 04-12 | SP | F | 3.5 |
| 04-13 | SP | F | 4.5 |
| 04-15 | A | F | 3.5 |
| 04-16 | IP | F | 7.5 |
| 04-18 | IP | F | 6.5 |
| 04-19 | IP | F | 2.5 |
| 04-20 | SP | F | 4.5 |
| 04-22 | DF | F | 7.5 |
| 04-23 | GM | F | 6.5 |
| 04-24 | NTM | M | 6.5 |
| 04-25 | A | F | 5.5 |
| 04-26 | MG | M | 4.5 |
| 04-27 | A | F | 3.5 |
| 05-01 | MG | M | 8 |
| 05-02 | MG | M | 5 |
| 05-03 | MG | M | 11 |
| 05-05 | NTM | M | 6 |
| 05-06 | NTM | M | 6 |
| 05-07 | NTM | M | 2 |
| 05-08 | MG | M | 12 |
| 05-09 | SM | M | 5 |
| 05-10 | SM | M | 9 |
| 05-11 | STM | M | 3 |
| 05-12 | STM | M | 8 |
| 05-13 | NTM | M | 6 |
| 05-14 | NTM | M | 6 |
| 05-15 | NTM | M | 2 |
| 05-16 | IP | F | 8.5 |
| 05-17 | IP | F | 5.5 |
| 05-18 | SP | F | 5.5 |
| 05-19 | SP | F | 8.5 |
| 05-20 | GM | F | 3.5 |
| 05-21 | GM | F | 4.5 |
| 05-23 | DF | F | 2.5 |
| 05-24 | DF | F | 2.5 |
| 06-01 | RC | F | 1.5 |
| 06-02 | WW | M | 4.5 |
| 06-03 | RC | F | 5.5 |
| 06-04 | MD | F | 3.5 |
| 06-05 | WW | M | 11.5 |
| 06-06 | WW | M | 9.5 |
| 06-07 | MB | F | 6.5 |
| 06-08 | EW | M | 6.5 |
| 06-09 | VP | F | 2.5 |
| 06-10 | SS | F | 3.5 |
| 06-11 | SS | F | 13.5 |
| 06-12 | SS | F | 10.5 |
| 06-13 | EW | M | 8.5 |
| 09-01 | CM | M | 6 |
| 09-02 | CM | M | 11 |
| 09-03 | MG | M | 8 |
| 09-04 | MG | M | 10 |
| 09-05 | SM | M | 6 |
| 11-01 | SM | M | 6.5 |
| 11-03 | SM | M | 7.5 |
| 11-04 | CM | M | 8.5 |
| 11-05 | CM | M | 6.5 |


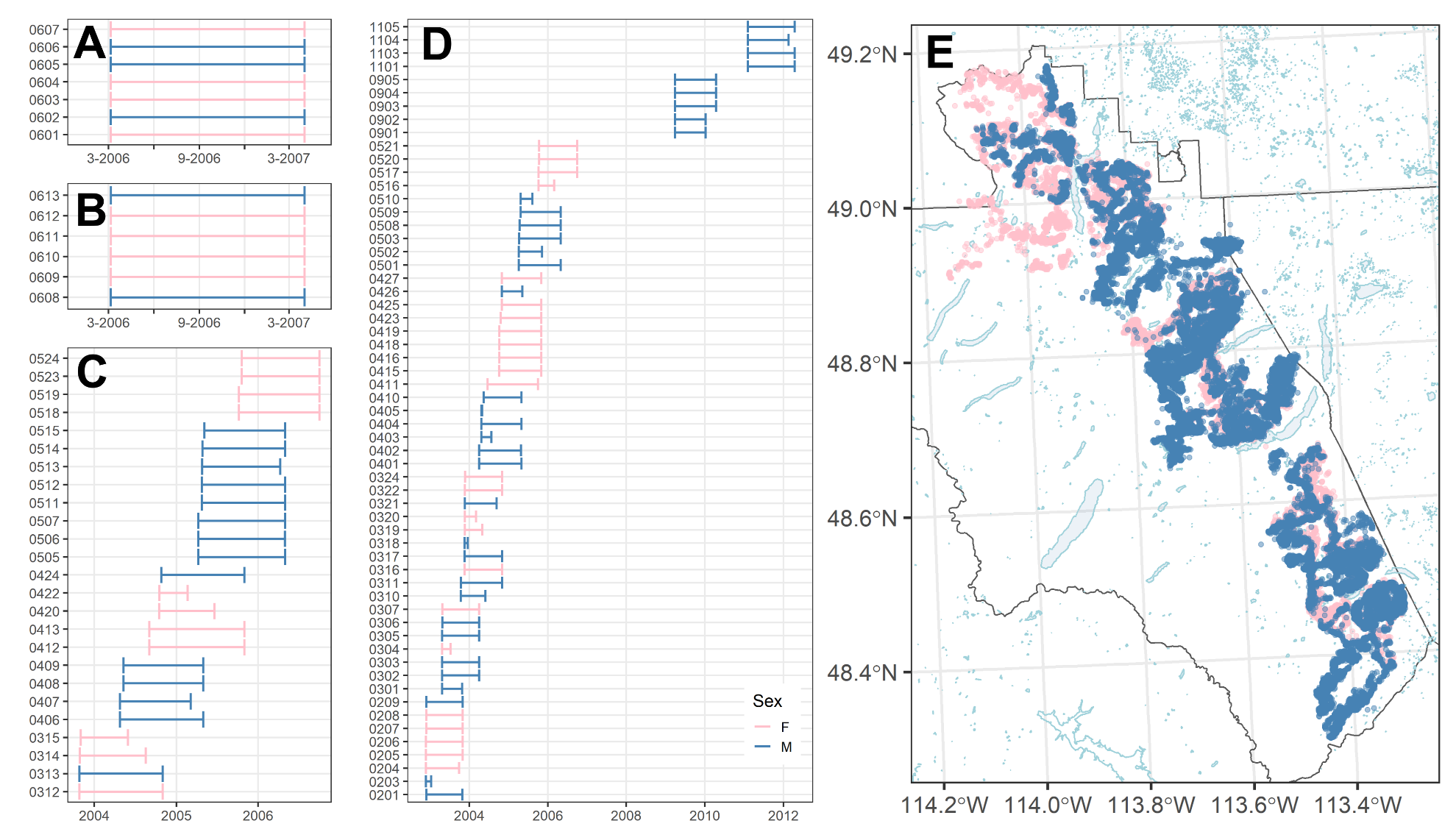


**Figure S1.** Collared bighorn sheep (*Ovis canadensis*) in the Waterton-Glacier International Peace Park during 2002-2012. Duration of active collars in (A) West Waterton Lakes National Park, (B) East Waterton Lakes National Park, (C) South Glacier area, and (D) North Glacier area. (E) Distribution of GPS locations across the study area.

**
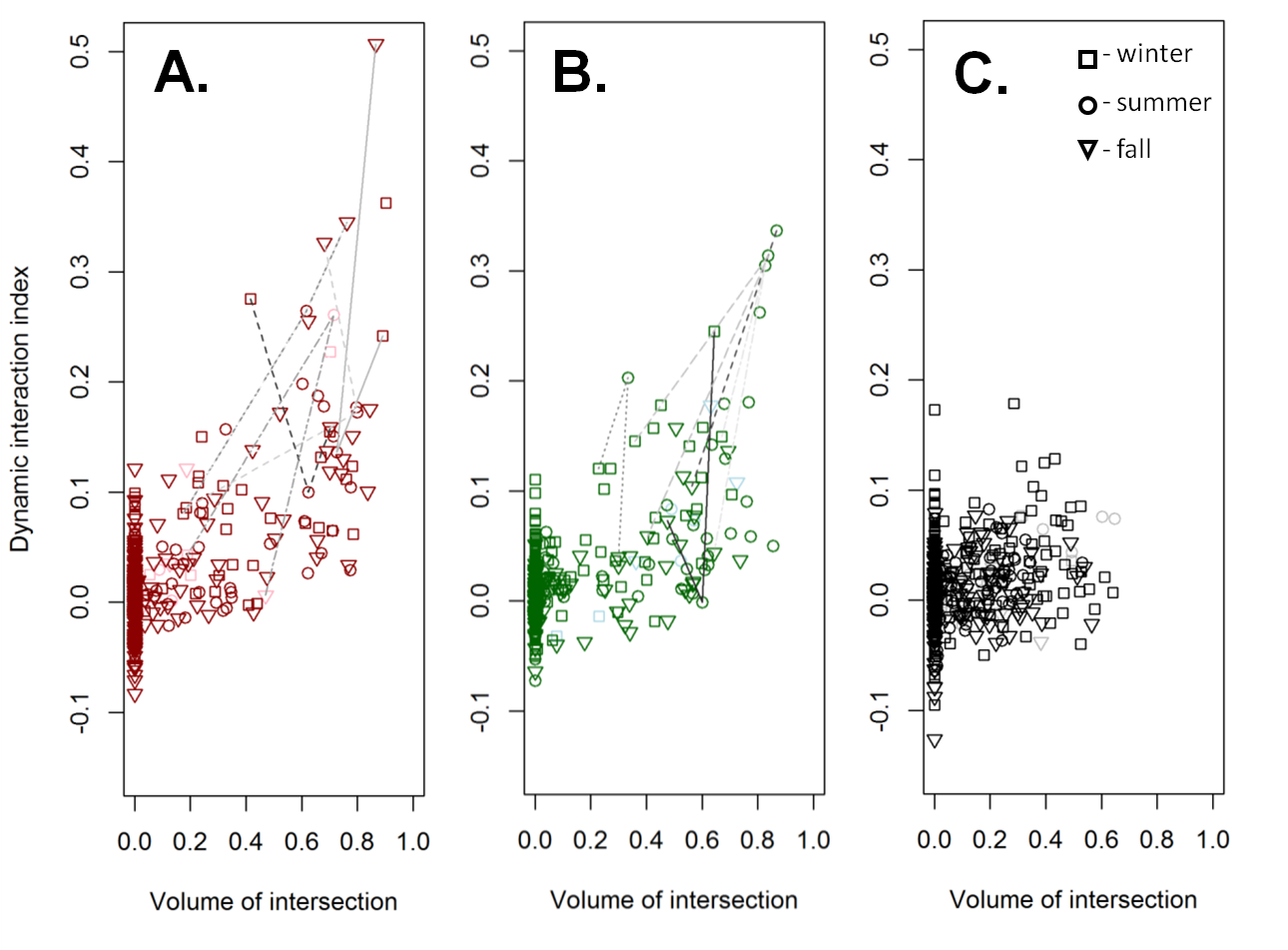
**

**Figure S2.** Correlation of movements of bighorn sheep dyads according to space-use overlap (volume of intersection) by sex. A) Female-female dyads, B) male-male dyads, and C) male-female dyads. Gray lines indicate the same dyad in different seasons. Lighter colors indicate yearling dyads, darker colors indicate adult dyads. Different line types used to help distinguish which points belong to the same dyad.


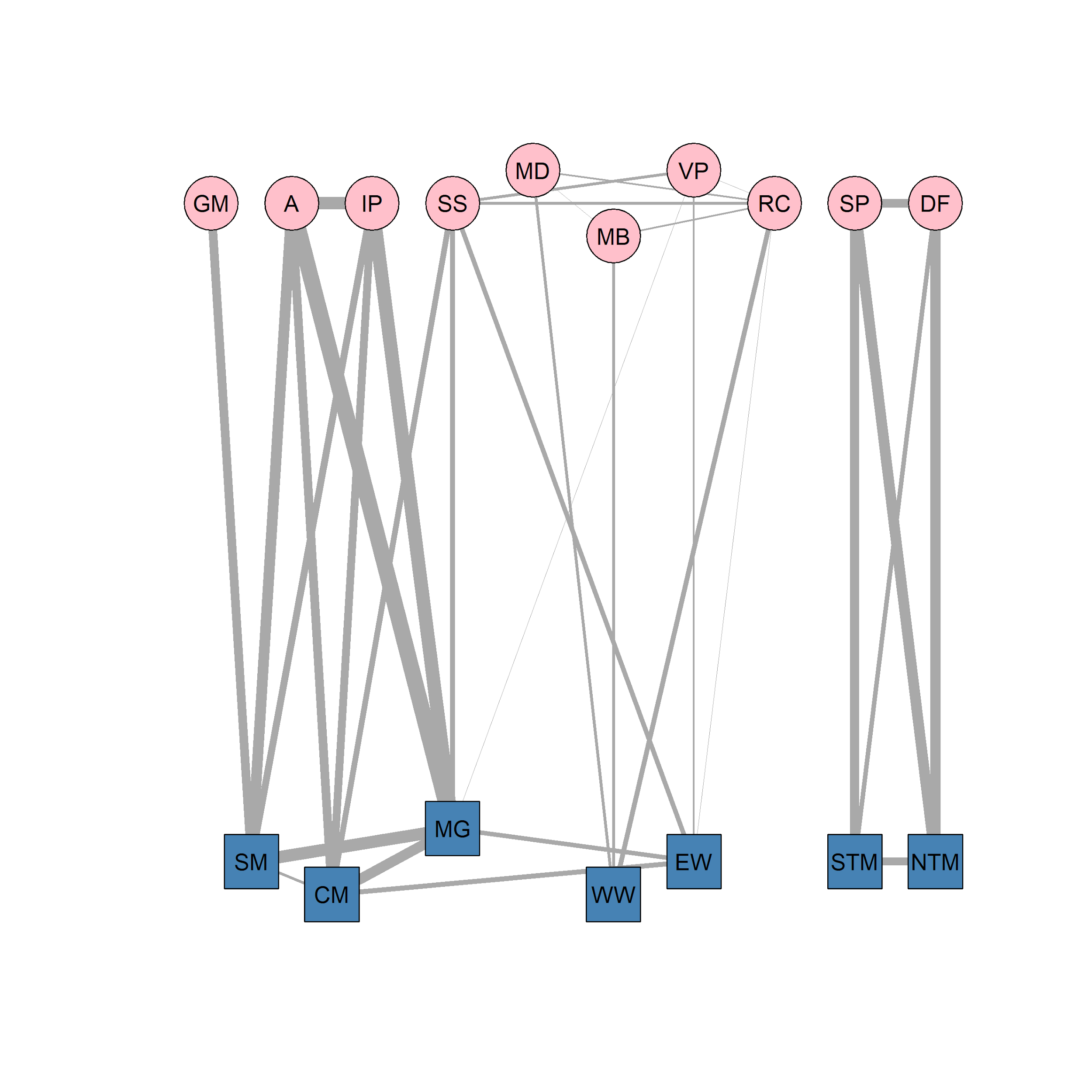


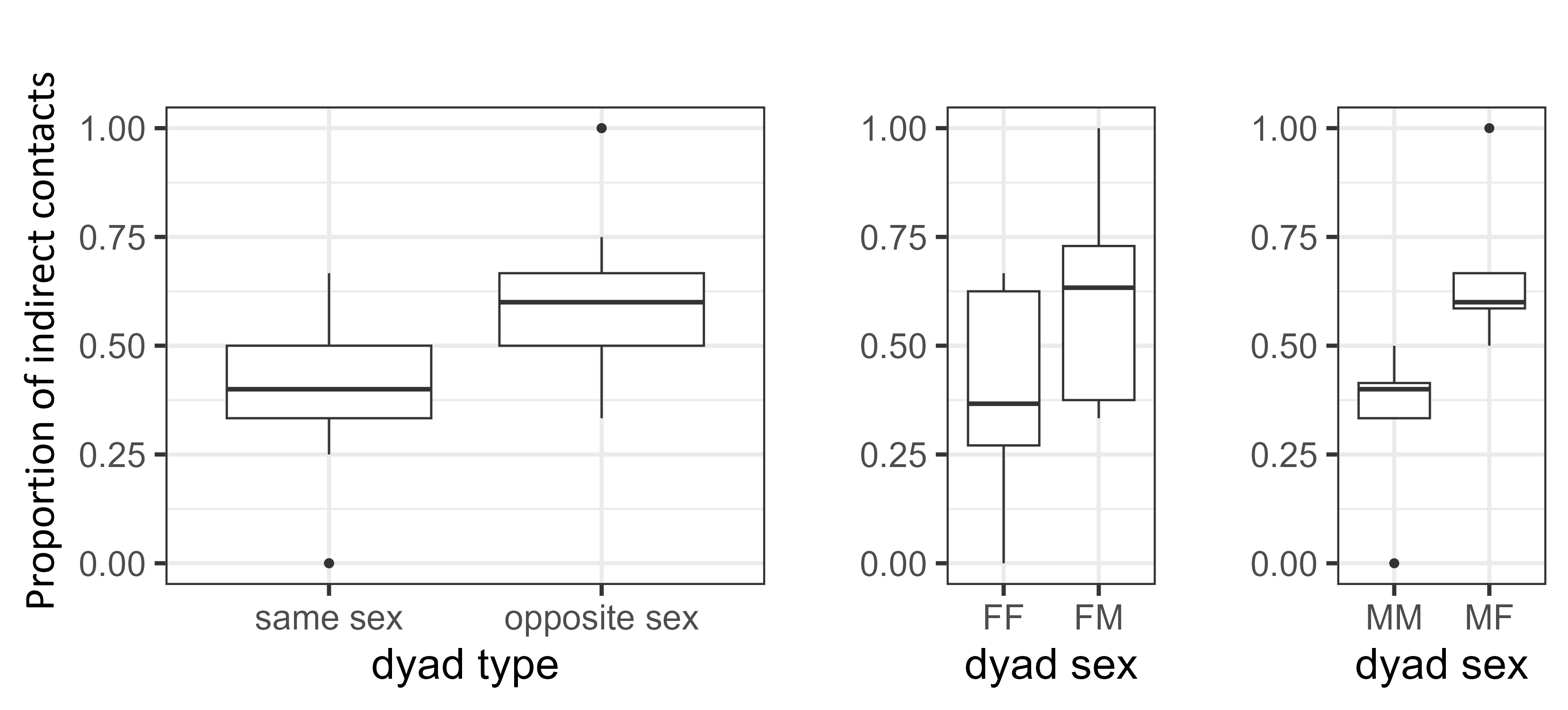


**Figure S3.** (A) Indirect contact network structure of bighorn sheep (*Ovis canadensis*) social groups (male = square, female = circle) and (B) comparison of indirect contacts among social groups of the same sex and opposite sex in Waterton-Glacier International Peace Park during 2002 – 2012. Indirect contacts defined as GPS locations < 25 m apart. Social groups are named after local landmarks (starting with the leftmost group, females then males): GM = Goat Mountain, A = Ahern, IP = Iceberg-Ptarmigan, SS = Sofa-Sarcee, MD = Dungarvan, MB = Blakiston, VP = Vimy Peak, RC = Richards, SP = Scenic Point, DF = Dry Fork, SM = St. Mary, CM = Chief Mountain, MG = Many Glacier, WW = West Waterton, EW = East Waterton, STM = South Two Medicine, and NTM = North Two Medicine.


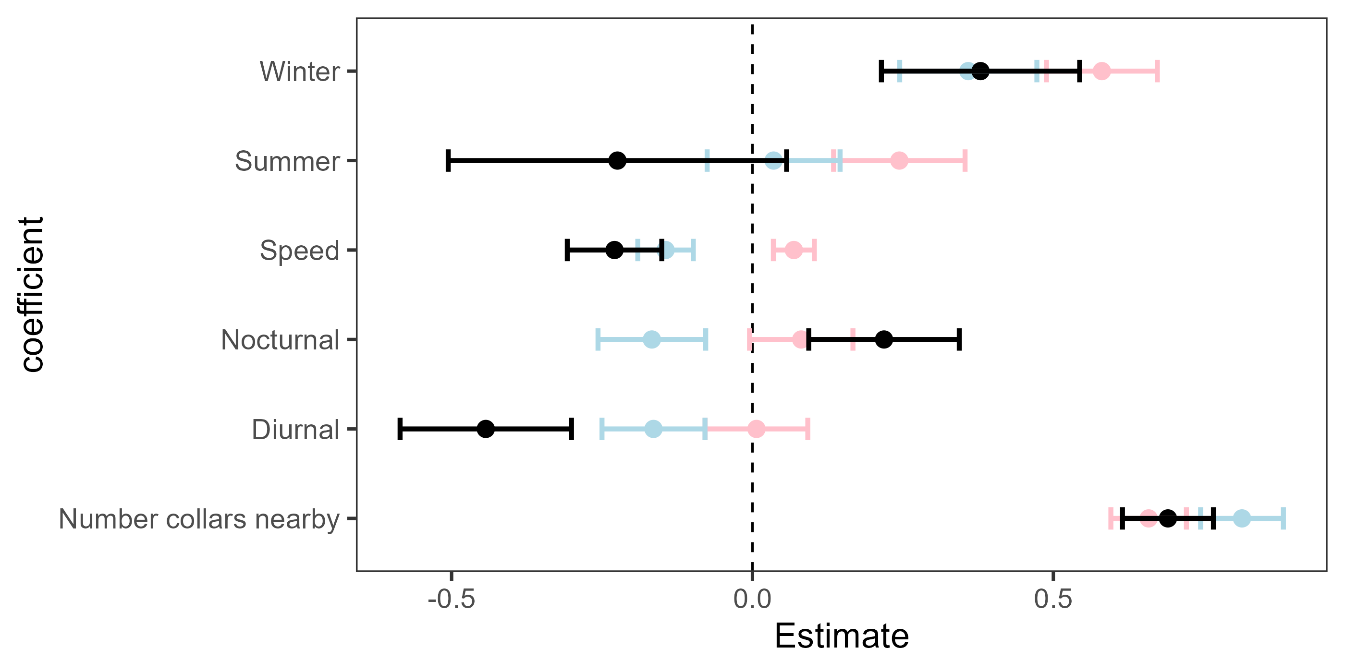


**Figure S4.** Variables from best model describing the timing of when contacts occurred between male-only (blue), female-only (pink), and male-female (black) bighorn sheep (*Ovis canadensis*) dyads in the Waterton-Glacier International Peace Park during 2002 – 2012. Note that time of day and season were categorical variables. Crepuscular hours were the reference category for nocturnal and diurnal categories and the fall season was the reference category for summer and winter.

Resources


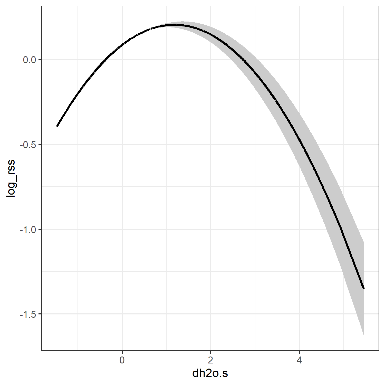

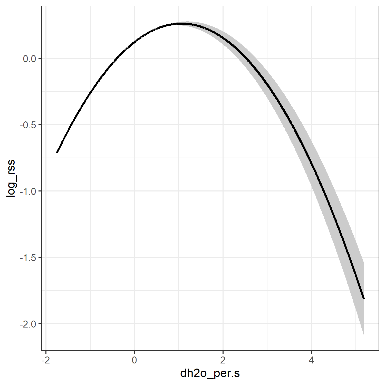

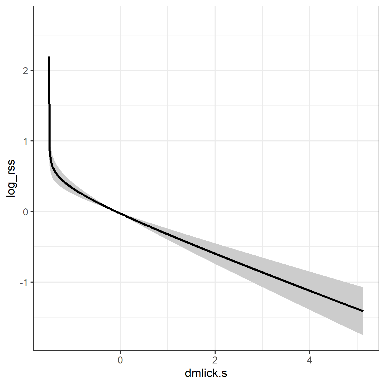

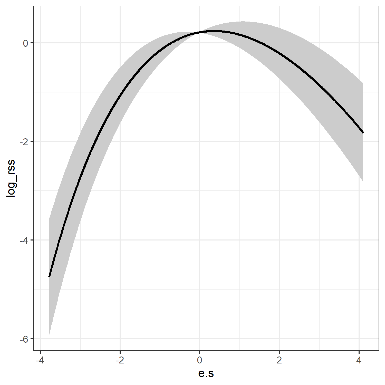

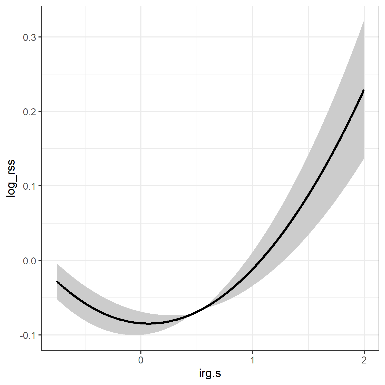

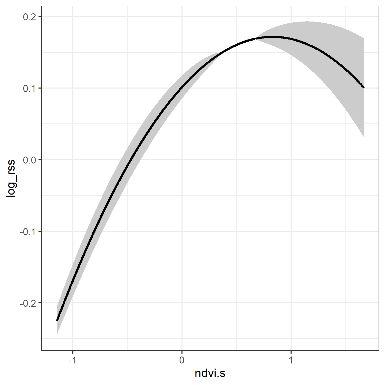

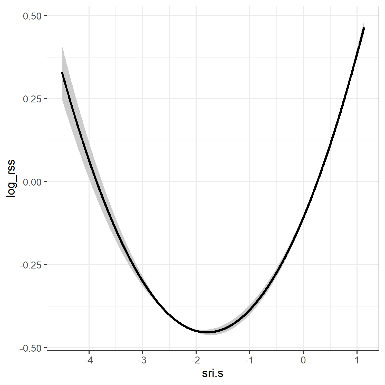

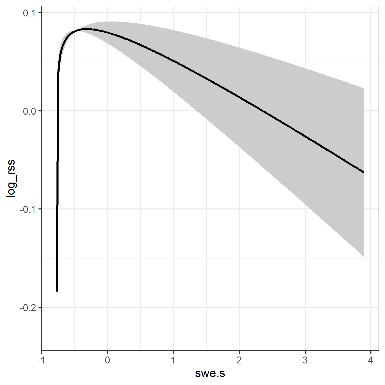


Survival


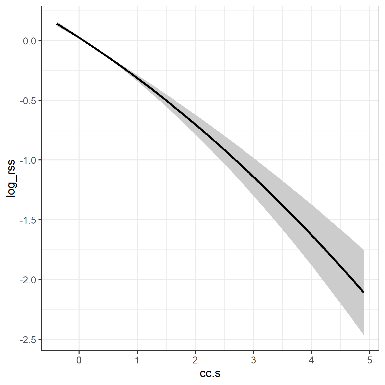

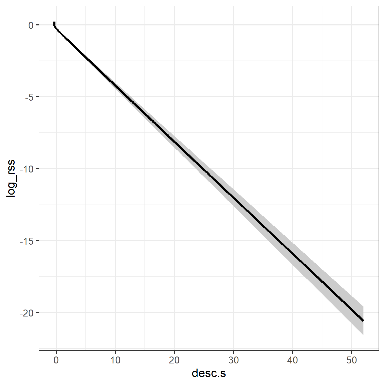

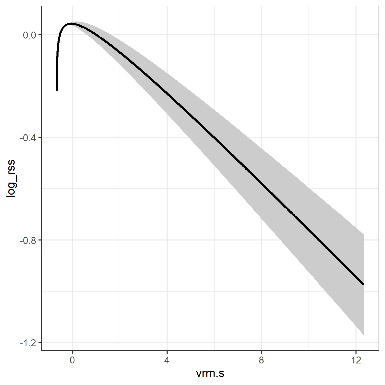


Disturbance


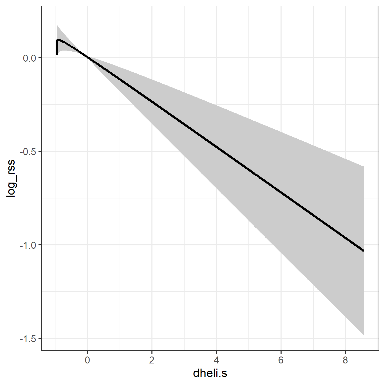

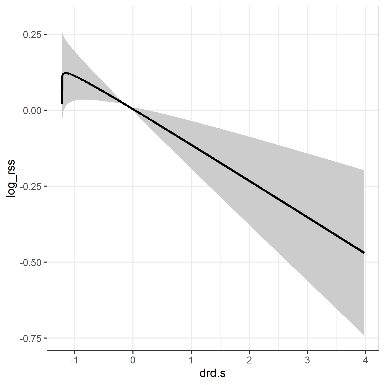

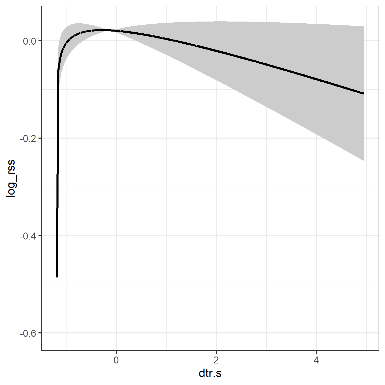

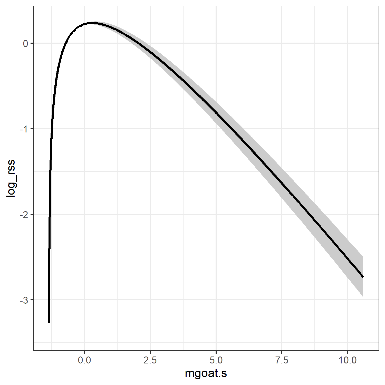


**Figure S5.** Predicted probabilities of use from univariate models of step-selection functions for male and female bighorn sheep (*Ovis canadensis*) in the Waterton-Glacier International Peace Park during 2002 – 2012. Ribbons indicate standard errors.


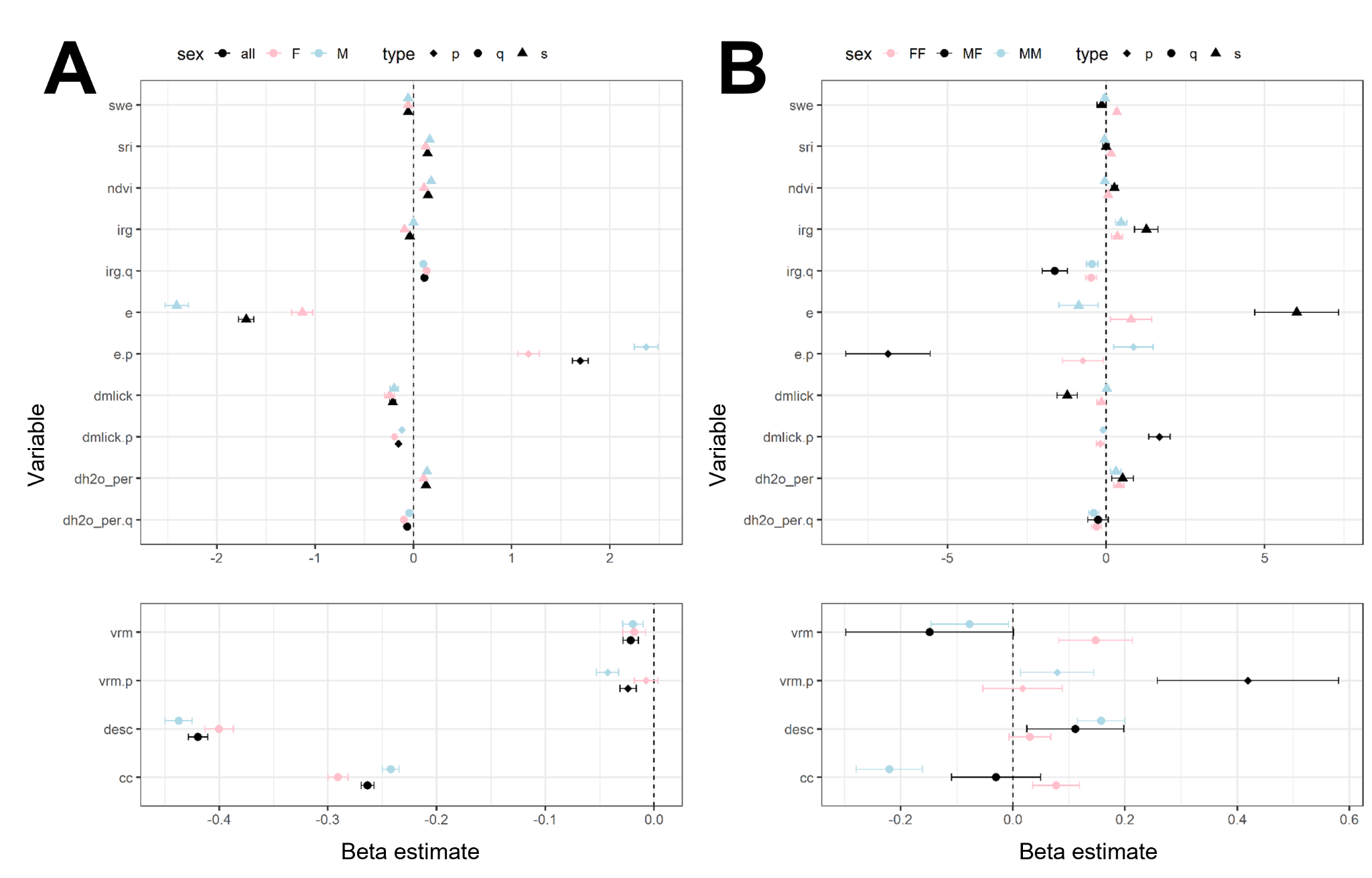


**Figure S6.** A) Global habitat selection models for males (blue), females (pink), and male and female (black) bighorn sheep (*Ovis canadensis*) and B) global contact models for male-only (blue), female-only (pink), and male-female (black) dyads of bighorn sheep in the Waterton-Glacier International Peace Park during 2002 – 2012. Variables are grouped by category: resource variables in top section, survival variables in the bottom section. Error bars represent standard errors. Shapes represent transformations to variables: pseudo-threshold (p; diamond), quadratic (q; circle), and centered and scaled (s; triangle).

Resources


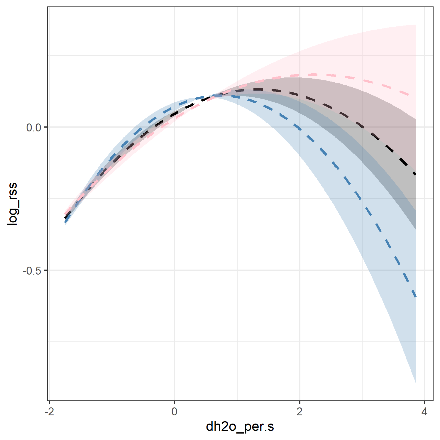

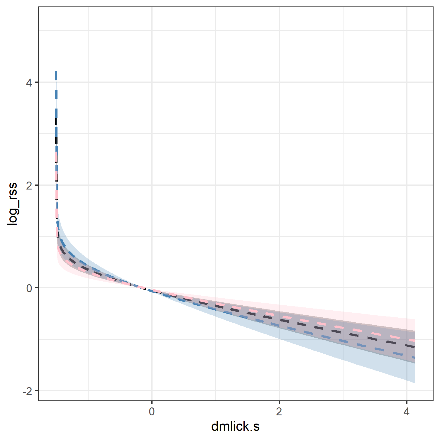

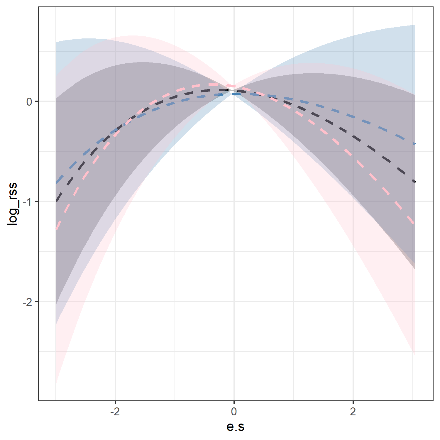

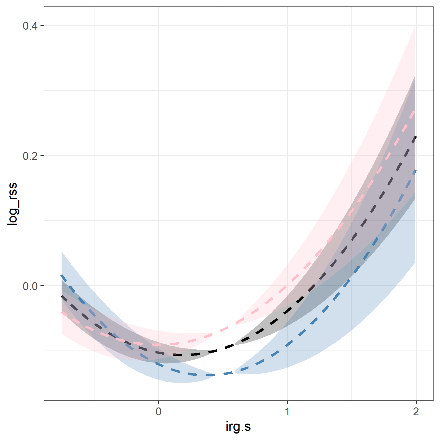

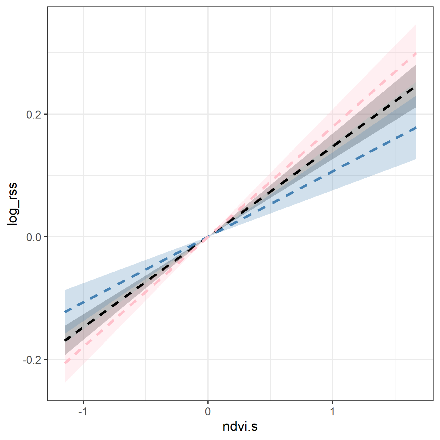

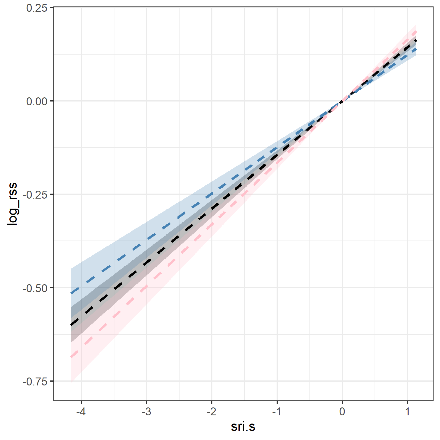

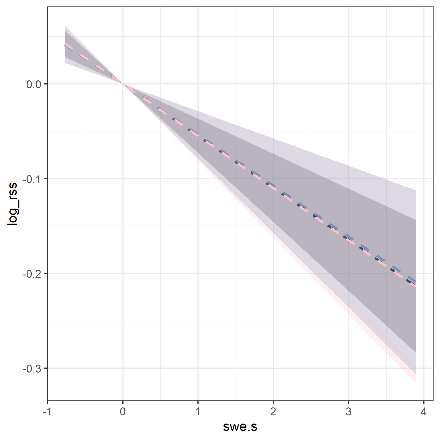


Survival


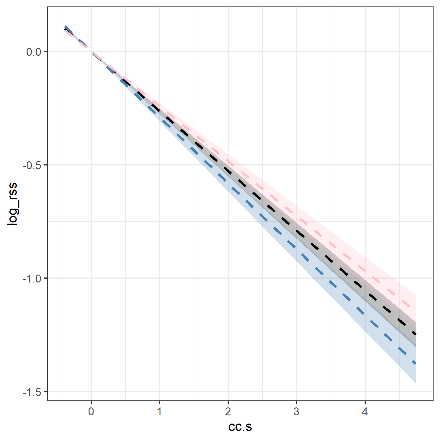

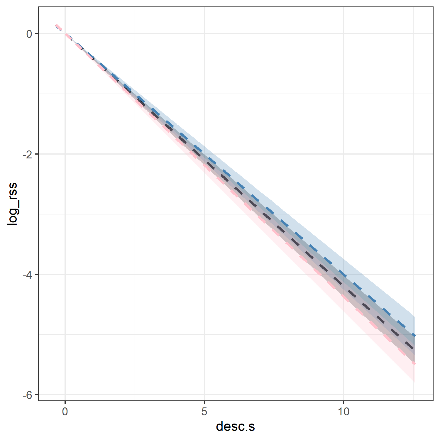

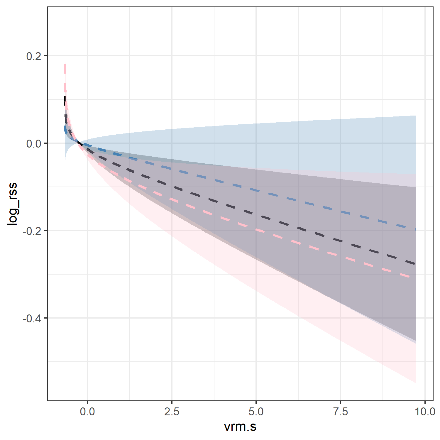


**Figure S7.** Predicted probabilities of use for global models of discrete-choice step-selection functions for male-only (blue), female-only (pink), and male and female (black) bighorn sheep (*Ovis canadensis*) in the Waterton-Glacier International Peace Park during 2002 – 2012. Ribbons indicate standard errors.


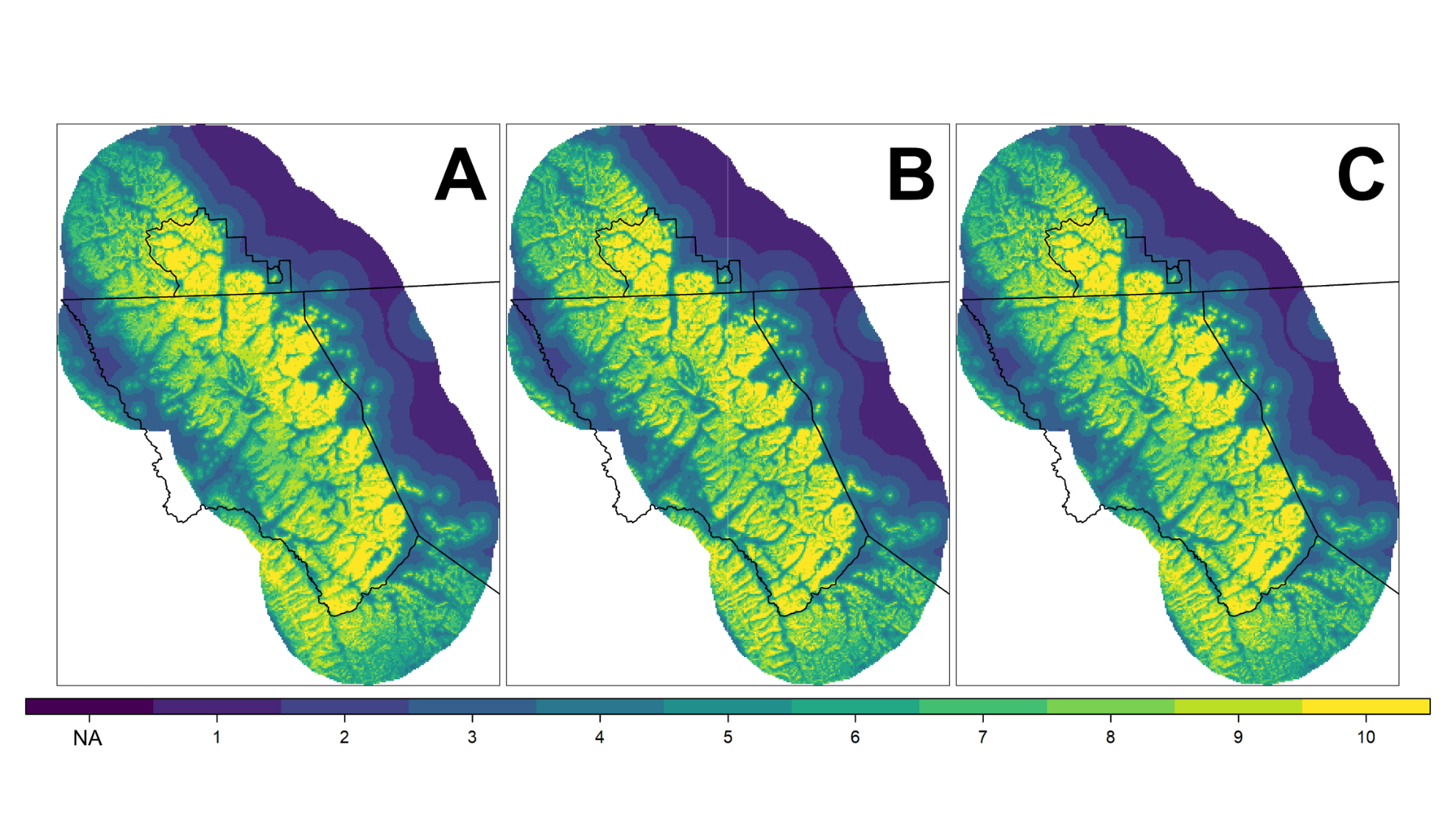


**Figure S8.** Predicted overall resource selection (1 = least likely to be used, 10 = most likely to be used), modeled as discrete-choice step-selection model. Habitat use of (A) female-only, (B) male-only, and (C) male and female bighorn sheep (*Ovis canadensis*) in the Waterton-Glacier International Peace Park during 2002 – 2012.


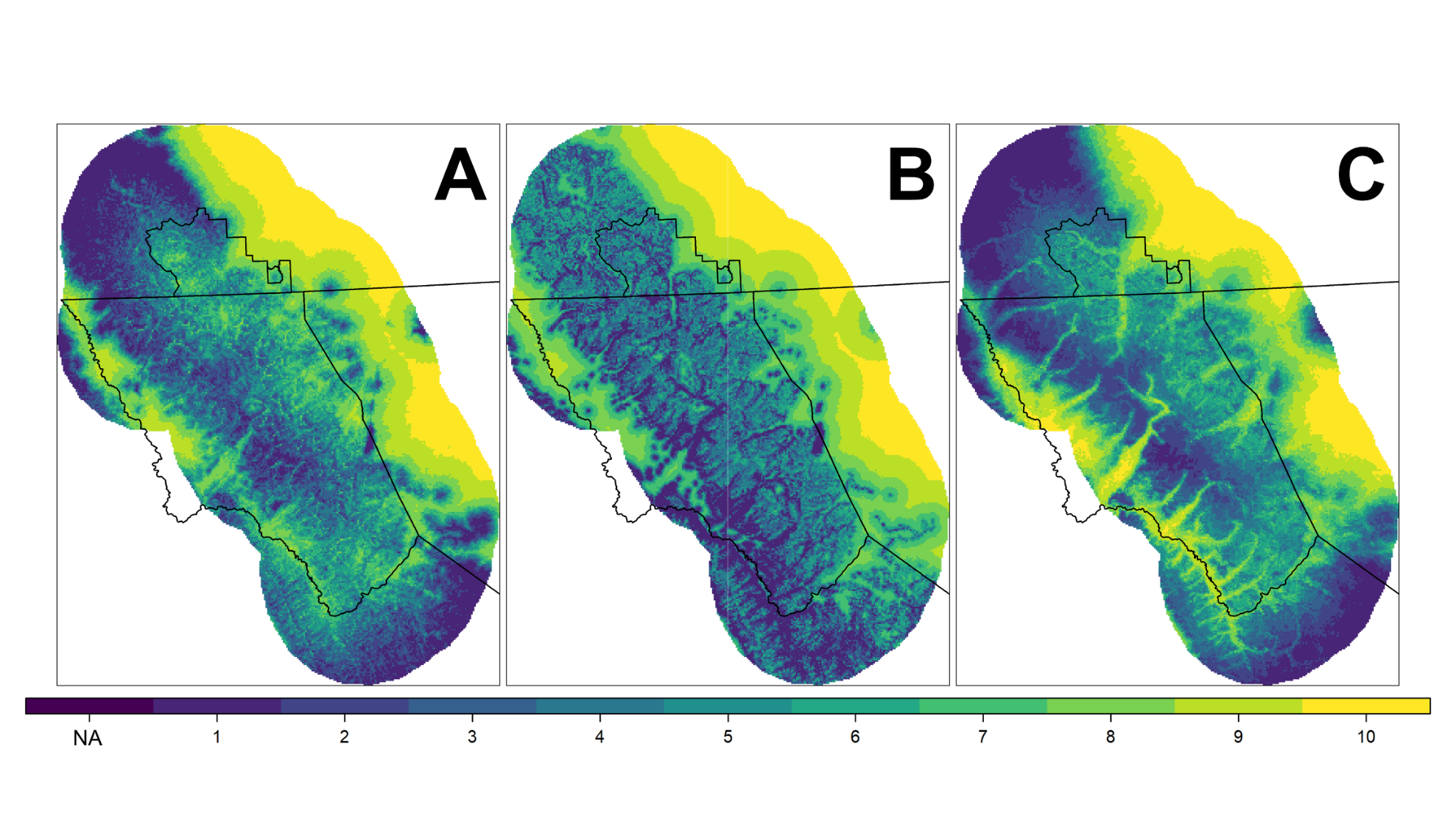


**Figure S9.**  Predicted resource selection of contacts, modeled as a resource selection function model (1 = least likely to contact, 10 = most likely to contact). Contacts between (A) female-female, (B) male-male, and (C) male-female dyads of bighorn sheep (*Ovis canadensis*) in the Waterton-Glacier International Peace Park during 2002 – 2012.

Resources


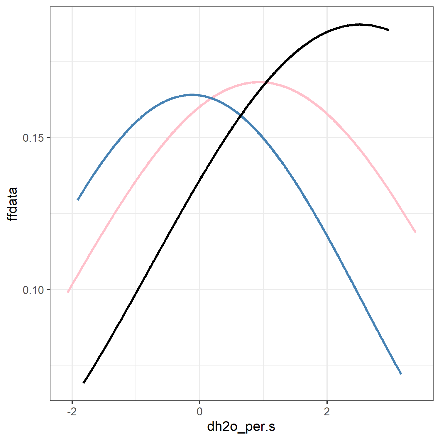

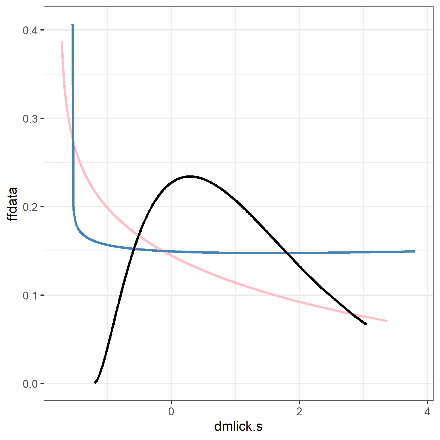

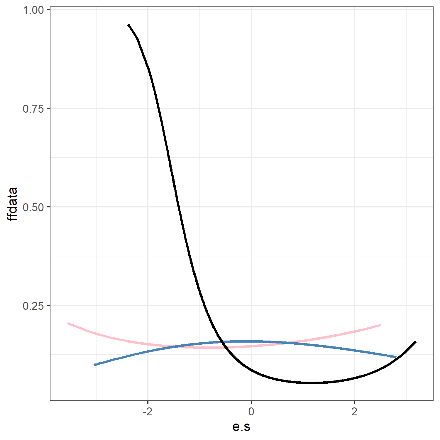

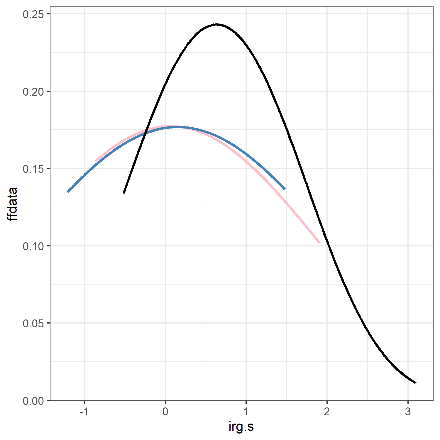

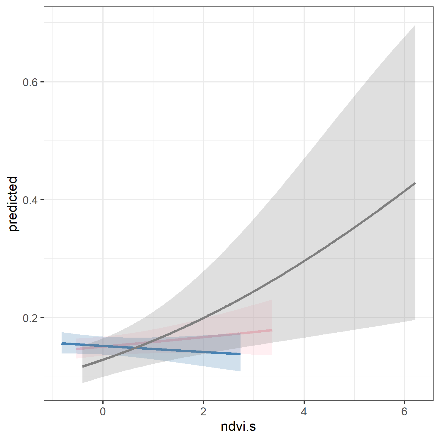

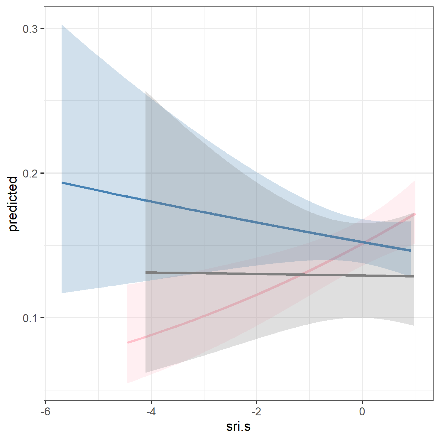

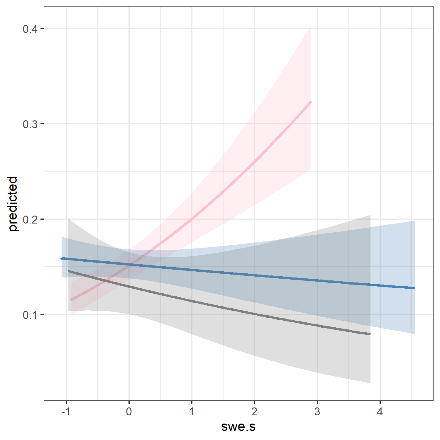


Survival


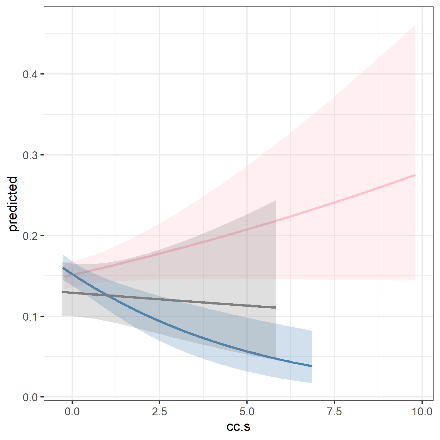

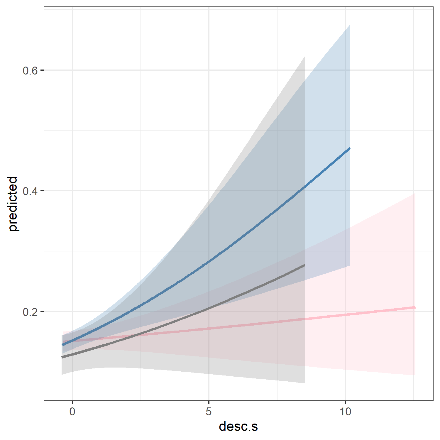

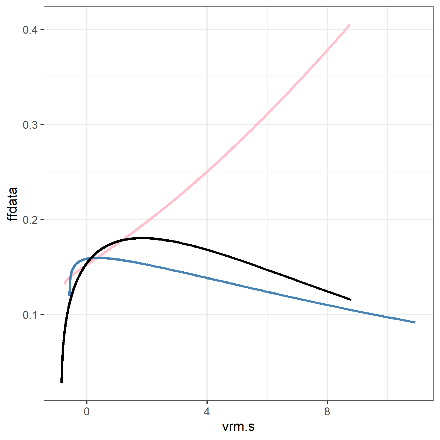


**Figure S10.**  Predicted probabilities of use from global models for contact resource selection functions for male-only (blue), female-only (pink), and male-female (black) dyads of bighorn sheep (*Ovis canadensis*) in the Waterton-Glacier International Peace Park during 2002 – 2012. Ribbons indicate standard errors.


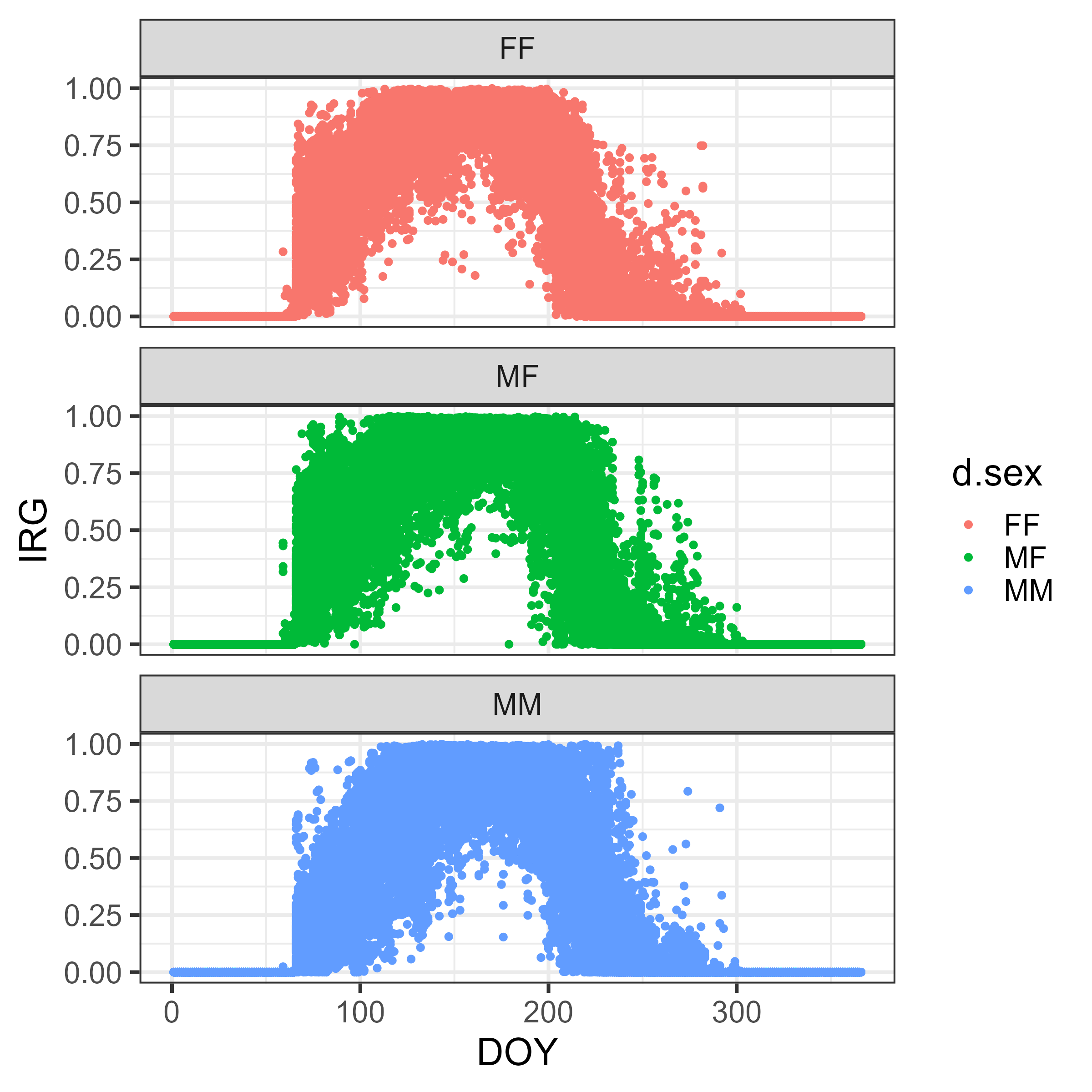


**Figure S11.** Day of year (DOY) vs. instantaneous rate of green up (IRG) of direct contact locations and locations where bighorn sheep (*Ovis canadensis*) where available for direct contact.
